# Enhanced *O*-GlcNAc modification induced by the RAS/MAPK/CDK1 pathway is required for SOX2 protein expression and generation of cancer stem cells

**DOI:** 10.1038/s41598-022-06916-y

**Published:** 2022-02-21

**Authors:** Masahiro Shimizu, Hiroshi Shibuya, Nobuyuki Tanaka

**Affiliations:** 1 Department of Molecular Oncology, Institute for Advanced Medical Sciences, Nippon Medical School, Bunkyo-ku, Tokyo 113-8602 Japan; 2 Department of Molecular Cell Biology, Medical Research Institute, Tokyo Medical and Dental University (TMDU), Bunkyo-ku, Tokyo 113-8510 Japan

**Keywords:** Cancer stem cells, Oncogenes, Growth factor signalling

## Abstract

Cancer stem cells (CSCs) have tumour initiation, self-renewal, and long-term tumour repopulation properties, and it is postulated that differentiated somatic cells can be reprogrammed to CSCs by oncogenic signals. We previously showed that oncogenic *HRAS*^*V12*^ conferred tumour initiation capacity in tumour suppressor p53-deficient (*p53*^*−/−*^) primary mouse embryonic fibroblasts (MEFs) through transcription factor NF-κB-mediated enhancement of glucose uptake; however, the underlying mechanisms of *RAS* oncogene-induced CSC reprogramming have not been elucidated. Here, we found that the expression of the reprogramming factor *SOX2* was induced by *HRAS*^*V12*^ in *p53*^*−/−*^ MEFs. Moreover, gene knockout studies revealed that *SOX2* is an essential factor for the generation of CSCs by *HRAS*^*V12*^ in mouse and human fibroblasts. We demonstrated that HRAS^V12^-induced cyclin-dependent kinase 1 (CDK1) activity and subsequent enhancement of protein *O*-GlcNAcylation were required for SOX2 induction and CSC generation in these fibroblasts and cancer cell lines containing *RAS* mutations. Moreover, the CDK inhibitor dinaciclib and *O*-GlcNAcylation inhibitor OSMI1 reduced the number of CSCs derived from these cells. Taken together, our results reveal a signalling pathway and mechanism for CSC generation by oncogenic *RAS* and suggest the possibility that this signalling pathway is a therapeutic target for CSCs.

## Introduction

Cancers are derived from cancer stem cells (CSCs), also known as cancer-initiating cells, that form tumours *in vivo*^1^. CSCs comprise a subset of tumour cells with tumour formation capacity, self-renewal properties, and long-term tumour repopulation activity^2,3^. The molecular mechanisms that support the reprogramming events leading to the generation of CSCs are considered to be similar to those of induced pluripotent stem cells (iPSCs), including the same regulatory factors and control mechanisms^4^. Indeed, reprogramming factors, including SRY-box 2 (SOX2), octamer-binding transcription factor 4 (OCT4, also known as POU5F1), Krüppel-like factor 4 (KLF4), and MYC (collectively known as OKSM transcription factors), and chromatin modifiers are required for establishment of iPSCs are associated with cancer generation^4,5^. It has been shown that transient expression of OKSM factors induced cancer development in various tissues in vivo and was accompanied by global changes in DNA methylation status^6^. Alterations of genes involved in the regulation of cell proliferation, such as oncogenes and tumour suppressor genes, may lead to cancer development^7,8^. Therefore, it is possible that analyses of reprogramming factors and cell growth regulators will lead to the elucidation of the mechanism of CSC generation. However, cancer develops because of the acquisition of successive hallmark cancer capabilities in a multistep pathogenic process^7,8^, and it is difficult to analyse the role of reprogramming factors in CSC generation using established cancer cell lines.

*P53* is the most frequently mutated gene in human cancer cells, and its gene product functions as a transcriptional activator of various target genes^9^. Accumulating evidence has shown that metabolic regulation by p53 is important for tumour suppression^10,11^. For example, p53 inhibits glycolysis, and the loss of p53 function changes the energy source from cellular respiration to glycolysis. This metabolic shift, known as the Warburg effect, favours tumour growth and is observed in many cancer cells^12^. In *p53*-deficient (*p53*^*−/−*^) mouse embryonic fibroblasts (MEFs), we previously found that oncogenic cell transformation and enhanced aerobic glycolysis were induced by an activating mutant of HRAS (HRAS^V12^) and were completely dependent on the transcription factor NF-κB^13^. Moreover, enhanced glycolysis-induced *O-*GlcNAcylation in *HRAS*^*V12*^-expressing *p53*^*−/−*^ MEFs further activated NF-κB^14^, suggesting that the NF-κB/glycolysis activation loop in *p53*^*−/−*^ cells is involved in enhanced energy production and CSC generation. The loss of p53 markedly increases the efficiency of somatic cell reprogramming to iPSCs, suggesting the existence of a p53-mediated barrier system that inhibits this reprogramming^15–18^. In various stem cell populations, enhanced glucose uptake is commonly observed and is critical for the acquisition and maintenance of stemness^19^. Moreover, direct *O*-GlcNAcylation of OCT4 and SOX2 has been shown to regulate pluripotency and reprogramming of embryonic stem cells^20^. In relation to these results, we found that IL-8 overexpression in colon and lung cancer cells enhanced *O*-GlcNAcylation, which was required for the production and maintenance of CSCs^21^. These results suggest that enhanced glycolysis and *O*-GlcNAcylation are involved in CSC generation and maintenance in p53-deregulated cells.

Gain of function mutations in *RAS* genes are frequently found in human cancers and represent markers of poor prognosis in patients with certain cancers^22,23^. The *RAS* family in humans comprises three genes, which are *HRAS*, *NRAS*, and *KRAS*. The proteins function as small GTPases. The activation of RAS is tightly regulated by guanine nucleotide exchange factors, which promote GTP-bound active states, and GTP-bound RAS interacts with and activates effector enzymes, such as RAF kinases. In the RAS-RAF cascade, activated RAF kinase phosphorylates and activates mitogen-activated protein kinase kinases 1 and 2 (MEK1 and MEK2), which leads to phosphorylation and activation of extracellular signal-regulated kinase (ERK) 1 and 2, also known as mitogen-activated protein kinase (MAPK) 3 and 1, respectively. The RAS-RAF-MAPK pathway mediates various biological functions, such as cell proliferation, survival, and differentiation, through a large number of ERK1/2 substrates that include transcription factors and cell cycle regulators^24^. Oncogenic mutations in the RAS-RAF-MAPK pathway contribute to various malignant phenotypes that include invasion, metastasis, relapse, and angiogenesis^5^.

Studies have shown that RAS-MAPK signalling promotes stemness and maintenance of CSCs^25^, but the underlying mechanisms have not been completely elucidated. Based on these studies, we analysed the expression of reprogramming factors in oncogenic *HRAS*-expressing *p53*^*−/−*^ MEFs as a simple model system to understand the molecular mechanism of *RAS*-induced CSC generation. As a result, we found that SOX2 induction by CDK1-mediated protein *O*-GlcNAcylation was essential for generation and maintenance of CSCs in p53-deficient cells.

## Results

### Oncogenic *RAS *induces *SOX2*-initiated CSC generation

**Figure 1:**
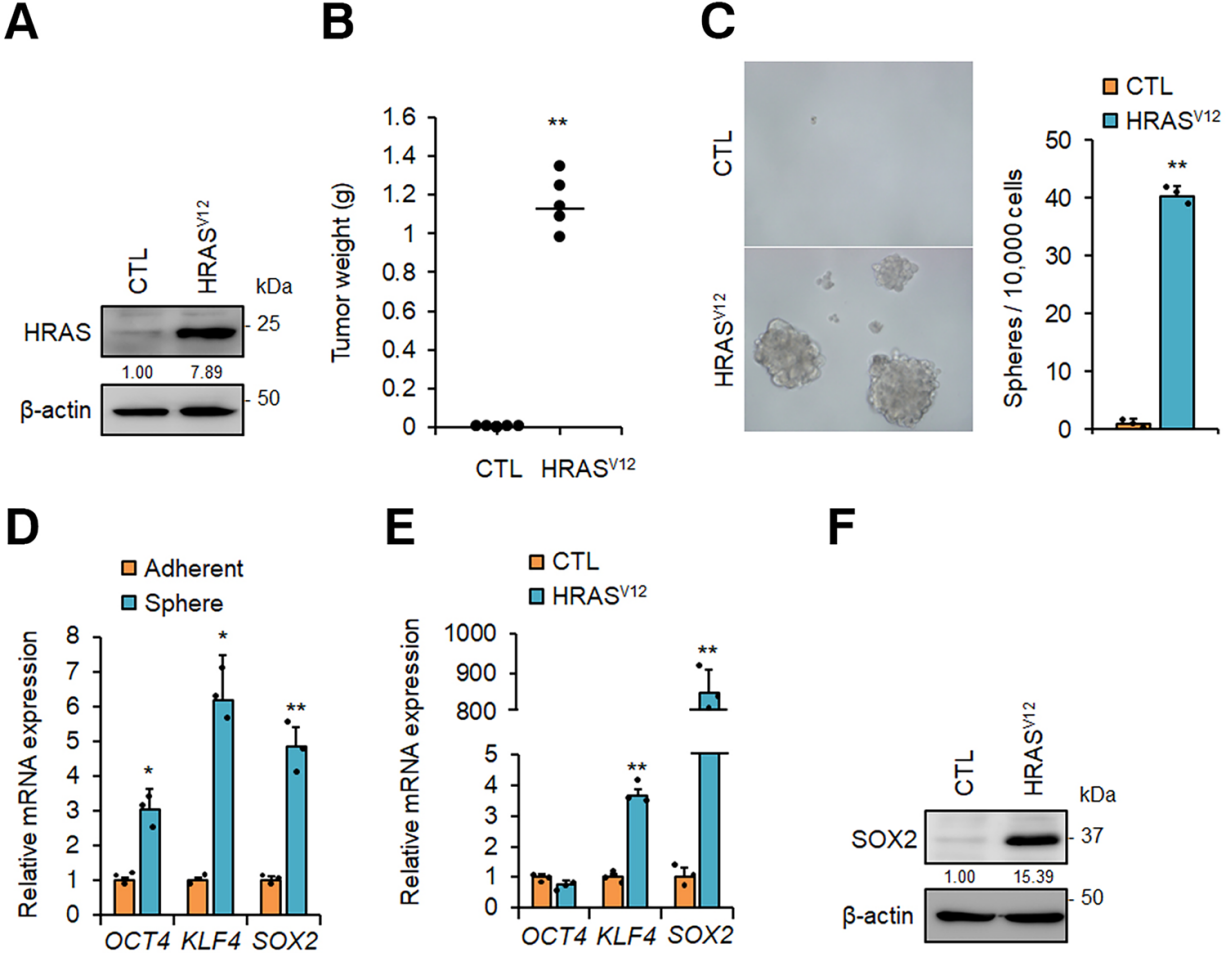
Oncogenic *RAS* induces *SOX2*-initiated CSC generation. (**A**) Vector only or the *HRAS*^*V12*^ mutant was transduced into *p53*^*−/−*^ mouse embryonic fibroblasts (MEFs) by retroviral infection for 2 days, and cells were selected with hygromycin for 3 days. Expression of the HRAS^V12^ mutant was confirmed by immunoblotting analysis. (**B**) *p53*^*−/−*^ MEFs (CTL) or *HRAS*^*V12*^-expressing *p53*^−/−^ MEFs (1 × 10^5^) were subcutaneously injected into immunodeficient mice (n = 5 per group). Tumour weights were measured 3 weeks after injection. **P < 0.01. (**C**) Sphere formation by *p53*^*−/−*^ MEFs (CTL) or *HRAS*^*V12*^-expressing *p53*^−/−^ MEFs. Representative images are shown on the left; the graph on the right shows quantification of the numbers of spheres counted per 10,000 cells. **P < 0.01. (**D**) qPCR analysis of the levels of the stem cell marker genes *OCT4*, *KLF4*, and *SOX2* in adherent cells or spheres from *HRAS*^*V12*^-expressing *p53*^−/−^ MEFs. The mRNA expression levels were normalised to β-actin. *P < 0.05, **P < 0.01. (**E**) mRNA expression levels of stem cell marker genes in *p53*^−/−^ MEFs (CTL) and *HRAS*^*V12*^-expressing *p53*^−/−^ MEFs. β-actin mRNA was used for normalisation. **P < 0.01. (**F**) Immunoblot of SOX2 protein expression in *p53*^−/−^ MEFs and *HRAS*^*V12*^-expressing *p53*^−/−^ MEFs. (**B**)–(**E**): Data are presented as the means ± SD of three independent experiments. Statistical analysis was performed with Student’s t-test. (**A**)and (**F**): The band intensity is provided under each band. Uncropped blot images are presented in Supplementary Fig. S5.

Previously, we reported that oncogenic *HRAS* (*HRAS* G12V mutant: *HRAS*^*V12*^) induced tumorigenic properties in *p53*^*−/−*^MEFs^26^, suggesting that *HRAS*^*V12*^ promoted the generation of CSCs. To confirm this hypothesis, we generated *HRAS*^*V12*^-expressing *p53*^−/−^ MEFs (Fig. 1A) and demonstrated that these cells formed tumours in nude mice (Fig. 1B). Previous studies have shown that CSCs display tumorigenic activity, and cancer cells with CSC-like properties will form spheres in low attachment culture conditions in medium containing growth factors^27,28^. As shown in Fig. 1C, approximately 0.4% of *HRAS*^*V12*^-expressing *p53*^−/−^MEFs developed spheres in low attachment plates. It has been reported that the reprogramming factors OCT4, KLF4, and SOX2 are important for generation and maintenance of CSCs and are used as CSC markers in various cancers^29–31^. Therefore, we analysed the mRNA expression levels of these factors and found that the sphere-forming cells exhibited enhanced expression of *OCT4*, *KLF4*, and *SOX2* compared with that of parental adherent cells (Fig. 1D). Moreover, although there may have been differences in reactivities between human and mouse antibodies, SOX2 protein expression in sphere-forming cells from *HRAS*^*V12*^-expressing MEFs was not significantly different from that observed in the human colon cancer line HCT116 (Supplementary Fig. S1A online). Furthermore, the protein expression level of OCT4 was 8.7-fold higher in sphere-forming cells from *HRAS*^*V12*^-expressing MEFs than that in HCT116. Therefore, these results suggested that sphere-forming cells from *HRAS*^*V12*^-expressing *p53*^−/−^MEFs were CSCs. We also compared expression levels of these genes between adherent *HRAS*^*V12*^-expressing *p53*^−/−^ MEFs cells and *p53*^−/−^ MEFs control cells. Interestingly, although *OCT4* expression was unchanged, the expression of *SOX2* was markedly enhanced in *HRAS*^*V12*^-expressing *p53*^−/−^ MEFs cells (Fig. 1E and F). These results suggest that the expression of *SOX2* as well as *KLF4* was induced by *HRAS*^*V12*^ expression in *p53*^−/−^MEFs, and *OCT4* was induced in the process of CSC reprogramming.

It has been shown that tumorigenic properties are efficiently induced by co-expression of oncogenes in primary rodent cells but not in human cells, suggesting a mechanistic difference between human and rodent oncogenesis^32^. As previously shown, *HRAS*^*V12*^ and simian virus 40 *large T antigen* (*SV40 LT*), which functionally inactivates p53 and the retinoblastoma tumour suppressor protein, were unable to induce tumorigenic properties in human cells^32^; SOX2 expression and sphere-forming activity were not induced by *p53* knockout (KO) or *HRAS*^*V12*^ expression in the human lung fibroblast cell line TIG-3 (Supplementary Fig. S1B and C online). However, tumorigenic properties were induced in TIG-3 cells by the combination of SV40 *T-ag*, *c-MYC*, and *HRAS*^*V12*^ (TIG-3–SMR cells)^33,34^, and sphere-forming cells were present in the TIG-3–SMR cell population (Supplementary Fig. S1D online). Notably, SOX2 expression was also elevated in TIG-3–SMR cells when compared with control TIG-3 cells (Supplementary Fig. S1E online). Moreover, in TIG-3-SM cells expressing only SV40 *T-ag* and *c-MYC*, SOX2 expression was not induced, and the SOX2 protein level was identical to that in control TIG-3 cells (Supplementary Fig. S1F online). These data indicated that RAS signalling was also required for the induction of SOX2 in TIG-3 cells. Therefore, these results suggest that the induction of SOX2 by oncogenic signalling is regulated differently between mice and humans. However, the difference in tissue origin cannot be ruled out.

### ***SOX2*** expression is essential for ***HRAS***^***V12***^-induced development of CSC properties

**Figure 2:**
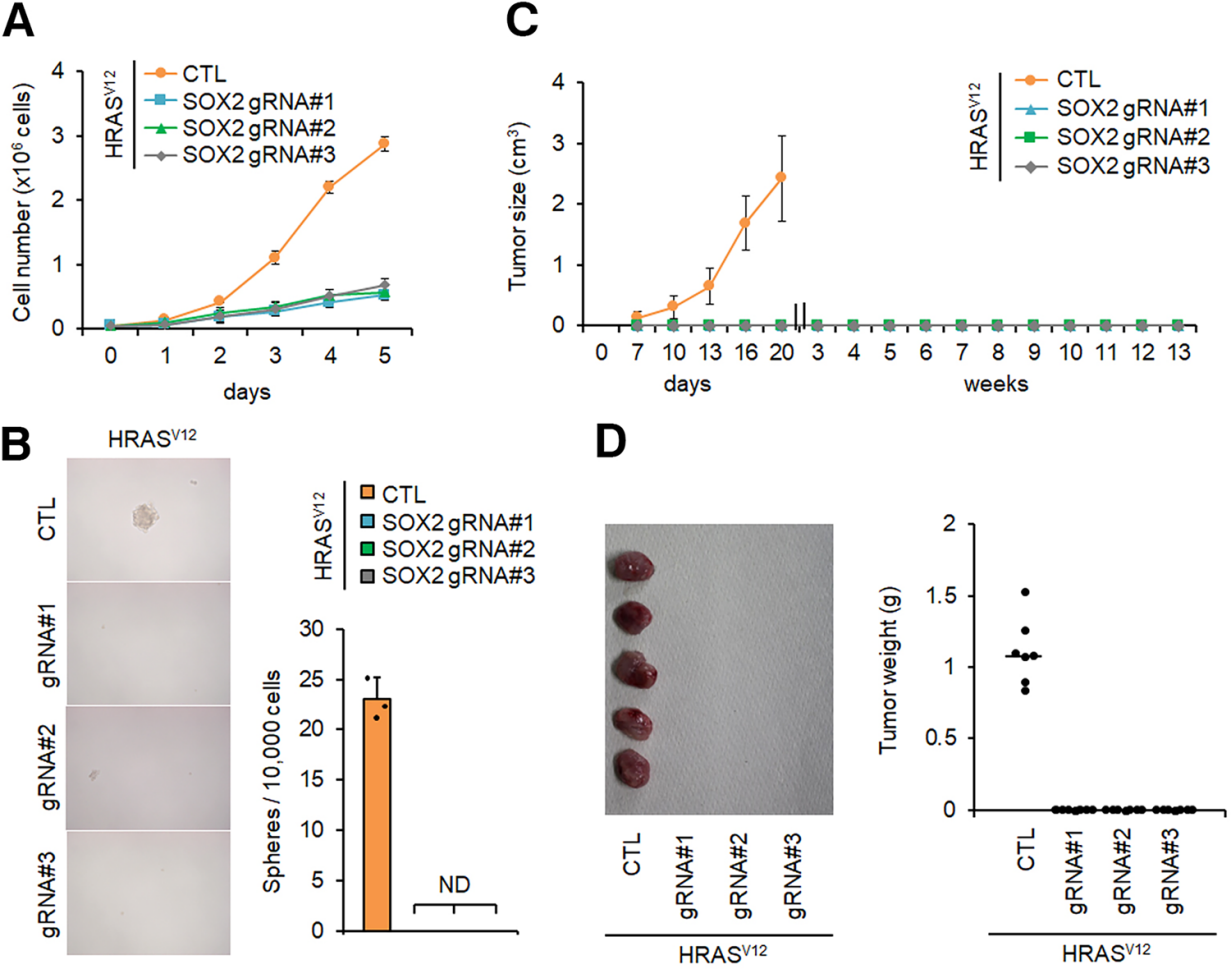
*SOX2* expression is essential for *HRAS*^*V12*^-induced development of CSC properties. (**A**) *SOX2* sgRNAs were co-expressed with CRISPR and Cas9 proteins in *HRAS*^*V12*^-expressing *p53*^−/−^ mouse embryonic fibroblasts (MEFs) by lentiviral infection for 2 days, and cells were selected with puromycin for 3 days. Growth curves for *HRAS*^*V12*^-expressing *p53*^−/−^ MEFs containing each *SOX2* gRNA or vehicle (CTL) are shown. (**B**) Sphere formation of SOX2-deficient *HRAS*^*V12*^-expressing *p53*^−/−^ MEFs. Representative images are shown on the left and quantification is shown on the right. ND, not detected. (**C**) and (**D**) Immunodeficient mice (n = 5) were subcutaneously injected with 1 × 10^5^ of *HRAS*^*V12*^-expressing *p53*^−/−^ MEFs containing each *SOX2* gRNA or vehicle (CTL). (**C**) Tumour sizes were measured every 3 days for 3 weeks and each week thereafter. (**D**) Tumours and tumour weights at 3 weeks post-injection for each mouse are indicated.

Studies have shown that *SOX2* functions as an oncogene, and expression is closely related to metastasis and relapse of many cancers^35,36^. Therefore, from these studies and the above results, it was possible that *SOX2* functioned as an initiation factor for CSC reprogramming. To test this hypothesis, we deleted the *SOX2* gene in *HRAS*^*V12*^-expressing *p53*^−/−^MEFs with three independent gRNAs using the CRISPR-Cas9 gene knockout system. Single cell-derived *SOX2* KO clones from each gRNA were isolated, and immunoblotting and quantitative PCR (qPCR) showed a lack of SOX2 expression in each clone (Supplementary Fig. S2A and B online). Inhibition of SOX2 expression was reported to attenuate cancer cell proliferation^37^. *SOX2* KO cells showed suppressed cell growth properties; however, proliferation was not completely inhibited (Fig. 2A). Next, we analysed the effect of *SOX2* KO on the CSC properties found in *HRAS*^*V12*^-expressing *p53*^−/−^MEFs. The appearance of sphere-forming cells and an increase in anchorage-independent colony-forming cells correlate with tumourigenicity^38^; however, these properties were not observed after *SOX2* KO (Fig. 2B and Supplementary Fig. S2C online). Furthermore, we analysed the effect of *SOX2* KO on tumour-initiating activity in vivo. *HRAS*^*V12*^-expressing *p53*^−/−^ MEFs with or without *SOX2* KO were subcutaneously injected into nude mice and tumour growth was monitored. Consistent with data presented in Fig. 1B, we confirmed tumour progression in mice injected with the *HRAS*^*V12*^-expressing *p53*^−/−^ MEFs (Fig. 2C). However, *SOX2* KO in these cells completely inhibited tumour development (Fig. 2C and D). Because the cell growth rate was suppressed in vitro by *SOX2* KO (Fig. 2A), we measured tumorigenic activity for an extended period; however, we did not detect tumour formation up to 13 weeks after injection (Fig. 2C). Moreover, forced expression of *SOX2* in *p53*^−/−^MEFs and *p53*^−/−^TIG-3 cells promoted sphere formation and tumour development (Supplementary Fig. S2D–H online). These results suggest that SOX2 expression is required for CSC generation in p53-deficient cells.

### SOX2 expression is induced by the RAF-MAPK pathway in ***HRAS***^***V12***^-expressing ***p53***^−/−^ MEFs

**Figure 3:**
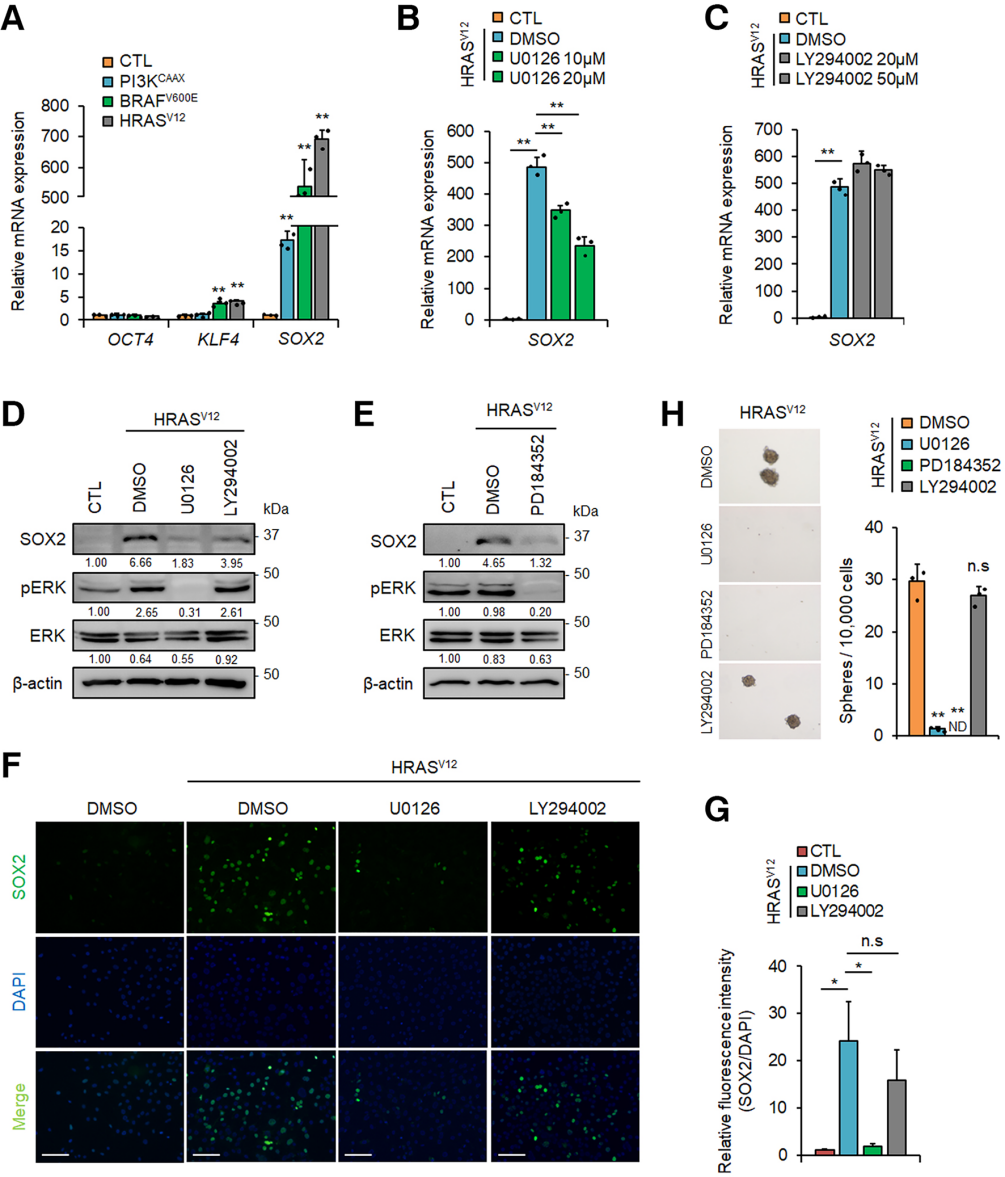
*SOX2* expression is induced by the RAF-MAPK pathway in *HRAS*^*V12*^-expressing *p53*^−/−^ mouse embryonic fibroblasts (MEFs). (**A**) Expression levels of *OCT4*, *KLF4*, and *SOX2* mRNA in *PI3K*^*CAAX*^-, *BRAF*^*V600E*^-, or *HRAS*^*V12*^-expressing *p53*^−/−^ MEFs. The levels of stem cell marker genes in these cells were compared with those in *p53*^−/−^ MEFs (CTL). **P < 0.01. (**B**) and (**C**), qPCR analysis of *SOX2* mRNA in *HRAS*^*V12*^-expressing *p53*^−/−^ MEFs treated with vehicle, U0126 (10, 20 µM), or LY294002 (20, 50 µM) for 24 h. **P < 0.01. (**D**) Immunoblotting analysis of SOX2 expression in *HRAS*^*V12*^-expressing *p53*^−/−^ MEFs treated with vehicle, U0126 (10 µM), or LY294002 (20 µM) for 24 h. (**E**) Expression levels of SOX2 protein in *HRAS*^*V12*^-expressing *p53*^−/−^ MEFs treated with vehicle or PD184352 (1 μM) for 24 h. (**F**) *HRAS*^*V12*^-expressing *p53*^−/−^ MEFs were stimulated with vehicle, U0126 (10 µM), or LY294002 (20 µM) for 24 h. The cells were stained with anti-SOX2 antibody (Green) and DAPI (Blue) and visualised by a fluorescence microscope under 200 × magnification. Scale bar = 100 µm. (**G**) Relative fluorescence intensity of SOX2 in panel F. n. s., not significant. *P < 0.05. (**H**) Sphere formation in *HRAS*^*V12*^-expressing *p53*^−/−^ MEFs cultured with vehicle (DMSO), U0126 (10 µM), PD184352 (1 µM), or LY294002 (20 µM) for 7 days. The numbers of spheres in each group were compared. ND, not detected; n. s., not significant. **P < 0.01. (**A**)–(**C**), (**G**), and (**H**): Data are presented as the means ± SD of three independent experiments. Statistical analysis was performed with Student’s t-tests. (**D**) and (**E**): The band intensity is provided under each band. Uncropped blot images are presented in Supplementary Fig. S5.

RAS proteins directly activate the downstream effectors RAF and PI3K followed by the downstream activation of MAPK and AKT pathways, respectively^24,39^. Therefore, we next examined whether these effectors were involved in promoting SOX2 expression. Constitutively active forms of RAF (BRAF^V600E^) and PI3K (PI3K^CAAX^) were stably expressed in *p53*^−/−^ MEFs. Although the increase in *SOX2* mRNA expression was relatively weakly in *PI3K*^*CAAX*^-expressing cells, the levels of *SOX2* and *KLF4* mRNA in *BRAF*^*V600E*^-expressing cells were similar to that in *HRAS*^*V12*^-expressing cells (Fig. 3A). These results suggest that *HRAS*^*V12*^ induces the expression of *SOX2* mRNA through RAF and its downstream effectors MEK/ERK. Furthermore, the expression of HRAS^V12^ enhanced the activating phosphorylation of ERK and both the mRNA and protein expression of SOX2. These enhancements were significantly reduced by the MEK inhibitor U0126 but not by the PI3K inhibitor LY294002 (Fig. 3B–D). Similar results were obtained after treatment with the MEK inhibitor PD184352 (Fig. 3E). Immunofluorescent staining confirmed that the fluorescence intensity of SOX2 was significantly reduced by the MEK inhibitor but not by the PI3K inhibitor (Fig. 3F and G). Additionally, the number of sphere-forming cells was reduced after treatment of *HRAS*^*V12*^-expressing *p53*^−/−^ MEFs with the MEK inhibitors (Fig. 3H). These results suggest that the RAF/MEK/ERK pathway is required for SOX2 induction and reprogramming of normal fibroblasts to CSCs.

### CDK1 activity is required for SOX2 induction and generation of CSCs in *RAS*-activated cells

**Figure 4:**
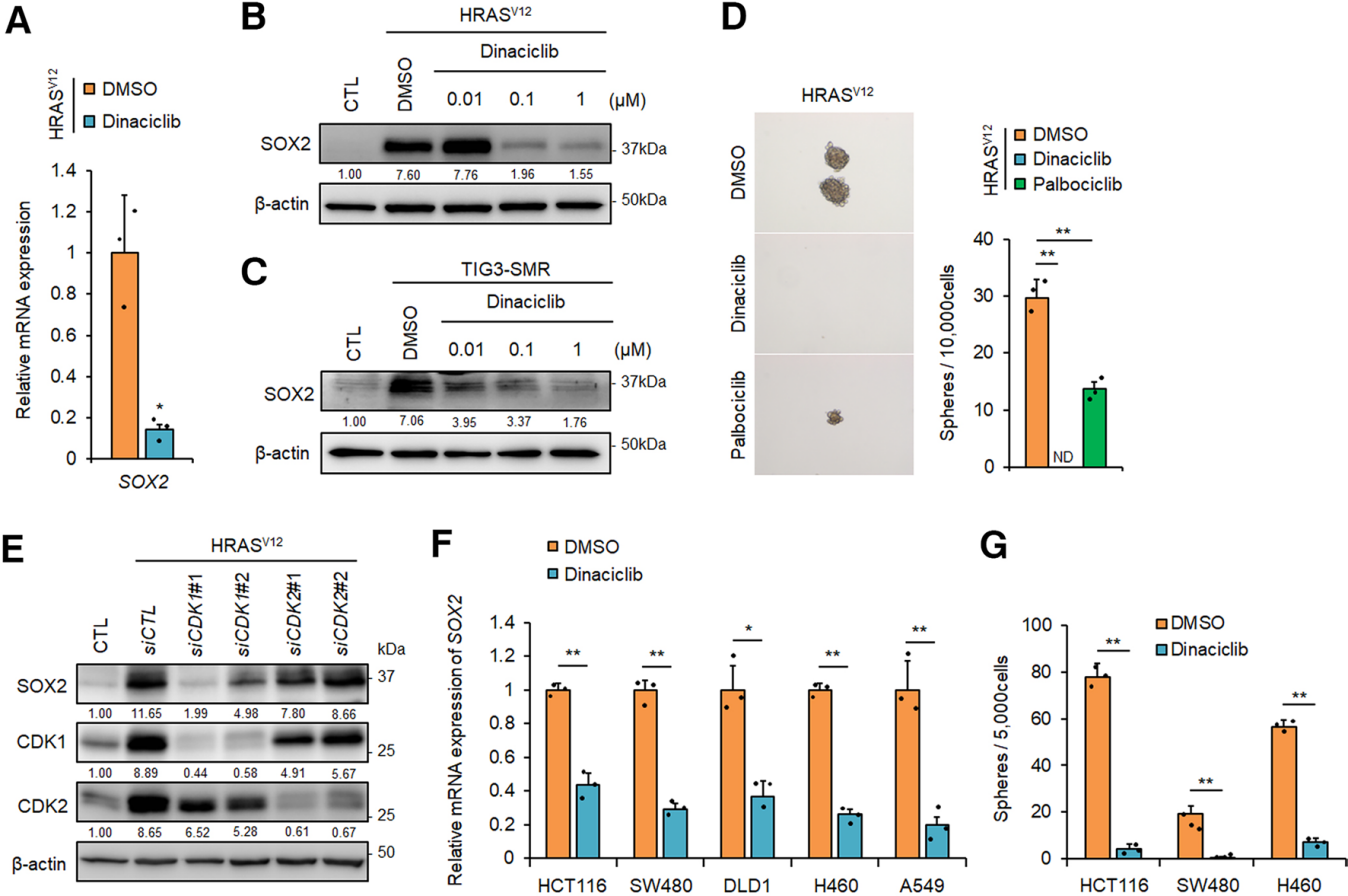
CDK1 activity is required for SOX2 induction and generation of CSCs in *RAS-*activated cells. (**A**) qPCR analysis of *SOX2* mRNA levels in *HRAS*^*V12*^-expressing *p53*^−/−^ MEFs treated with vehicle (DMSO) or dinaciclib (0.1 µM) for 24 h. *P < 0.05. (**B**) and (**C**) Western blot analysis of SOX2 expression in *HRAS*^*V12*^-expressing *p53*^−/−^ MEFs (**B**) and TIG-3–SMR cells (**C**) treated with vehicle or dinaciclib (0.01, 0.1, 1 µM) for 24 h. (**D**) Sphere formation of *HRAS*^*V12*^-expressing *p53*^−/−^ MEFs after 7 days in the presence or absence of dinaciclib (0.1 µM) or palbociclib (10 µM). Representative images are shown on the left, and quantification is shown on the right. ND, not detected. **P < 0.01. (**E**) Expression levels of SOX2 in *HRAS*^*V12*^-expressing *p53*^−/−^ MEFs following knockdown of CDK1 or CDK2 by siRNAs. (**F**) qPCR analysis of *SOX2* mRNA expression in HCT116, SW480, DLD1, H460, and A549 cancer cell lines treated with dinaciclib (0.1 µM) for 24 h. *P < 0.05, **P < 0.01. (**G**) Sphere formation assay of cancer cells in the presence or absence of dinaciclib (0.1 µM) for 7 days. **P < 0.01. (**A**), (**D**), (**F**), and (**G**): Data are presented as the means ± SD of three independent experiments. Statistical analysis was performed with Student’s t-tests. (**B**), (**C**), and (**E**): The band intensity is provided under each band. Uncropped blot images are presented in Supplementary Fig. S5.

Activation of ERK enhances expression of cyclin D1, which binds to and activates cyclin-dependent kinase 4 and 6 (CDK4/6) during the G1 phase of the cell cycle^40^. Aberrant regulation of the CDK4/6-cyclin D1 axis is associated with the development of metastatic melanoma and breast cancer^41,42^. From these findings, it is possible that *HRAS*^*V12*^ induced SOX2 expression through ERK-mediated CDK4/6 activation. To test this possibility, we analysed the effect of the CDK4/6 inhibitor palbociclib^43^ on SOX2 expression and confirmed that the expression of *SOX2* mRNA and protein levels were slightly reduced in *HRAS*^*V12*^-expressing *p53*^−/−^ MEFs after treatment with a high-dose of palbociclib (Supplementary Fig. S3A and B online). Similar results were obtained with TIG-3–SMR cells (Supplementary Fig. S3C online). These results suggest that CDK4/6 activity alone is insufficient for SOX2 induction. In addition to CDK4/6, the BRAF/MEK/MAPK pathway is also essential for some functions of CDK1 and CDK2^44–46^. Recent studies have reported a role for CDK1 and CDK2 in the maintenance of stem cell pluripotency and CSC-like properties in breast cancer^47–49^. These reports suggested that CDK1 and/or CDK2 are required for SOX2 induction. Therefore, we examined the effects of the CDK1/2 inhibitors dinaciclib^50^ and roscovitine^51^ on SOX2 expression. We found that *SOX2* mRNA and protein expression was strongly suppressed by dinaciclib (Fig. 4A and B) and roscovitine (Supplementary Fig. S3D and E online) in *HRAS*^*V12*^-expressing *p53*^−/−^ MEFs. Similar results for dinaciclib were observed in TIG-3–SMR cells (Fig. 4C). Moreover, the number of sphere-forming cells in *HRAS*^*V12*^-expressing *p53*^*−/−*^ MEFs was largely suppressed by dinaciclib but only partially suppressed by palbociclib (Fig. 4D), which correlated with suppression of *SOX2* expression levels (Fig. 4A and Supplementary Fig. S3A online). To further analyse whether CDKs were essential for SOX2 expression, we analysed SOX2 levels in *HRAS*^*V12*^-expressing *p53*^*−/−*^ MEFs after knockdown of *CDK1* or *CDK2* using siRNA. Immunoblot analysis showed that *CDK1*, but not *CDK2*, knockdown reduced the expression level of SOX2 protein (Fig. 4E). Treatment with dinaciclib, U0126, and PD184352, which suppressed the expression of SOX2 in this study, reduced the phosphorylation of CDK1^T161^, an indicator of CDK1 activation^52^ (Supplementary Fig. S4A–C online). These results indicate that CDK1 activity is required for SOX2 expression in *HRAS*^*V12*^-expressing *p53*^*−/−*^ MEFs. Additionally, we analysed the effect of dinaciclib on human cancer cell lines, including KRAS-mutated colon cancer (HCT116, SW480, and DLD1) and lung cancer (H460 and A549) cells. Dinaciclib suppressed *SOX2* mRNA expression in these cancer cells (Fig. 4F) and the number of sphere-forming cells from the HCT116, SW480, and H460 lines (Fig. 4G). These results suggest that CDK1-mediated SOX2-induction promotes the generation of CSCs in human cancer cells with RAS mutations.

### Enhanced *O*-GlcNAc modification induced by the RAS/MAPK/CDK1 pathway is required for SOX2 protein expression and generation of CSCs

**Figure 5:**
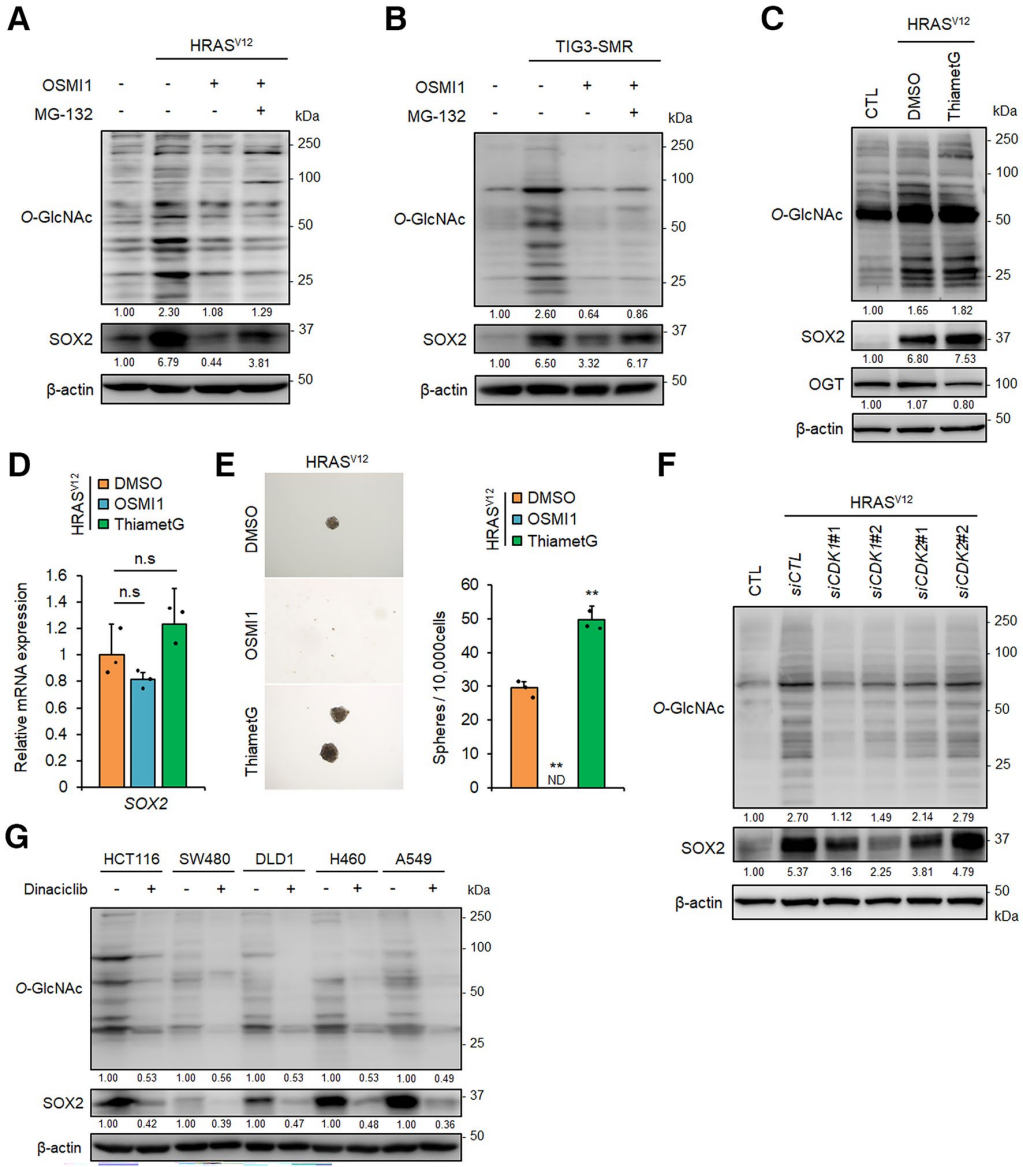
Enhanced *O*-GlcNAc modification induced by the RAS/MAPK/CDK1 pathway is required for SOX2 protein expression and generation of CSCs. (**A**) and (**B**) Immunoblot analysis of *O*-GlcNAc-modified proteins and SOX2 expression in *HRAS*^*V12*^-expressing *p53*^−/−^ mouse embryonic fibroblasts (MEFs) (**A**) and TIG-3–SMR cells (**B**) treated with vehicle or OSMI1 (50 µM) for 24 h in the presence or absence of MG-132 (20 μM) for 6 h. (**C**) Immunoblots of *O*-GlcNAc modified proteins and SOX2 expression in *HRAS*^*V12*^-expressing *p53*^−/−^ MEFs treated with vehicle or thiamet G (10 µM) for 24 h. (**D**) Expression levels of *SOX2* mRNA in *HRAS*^*V12*^-expressing *p53*^−/−^ MEFs cells treated with OSMI1 (50 µM) or thiamet G (10 µM) for 24 h. n. s., not significant. (**E**) Sphere formation assay of *HRAS*^*V12*^-expressing *p53*^−/−^ MEFs cultured with OSMI1 (50 µM) or thiamet G (10 µM) for 7 days. Representative images are shown on the left; the graph on the right shows quantification of the numbers of spheres counted per 10,000 cells. ND, not detected. **P < 0.01. (**F**) Expression levels of *O*-GlcNAc-modified proteins and SOX2 in *HRAS*^*V12*^-expressing *p53*^−/−^ MEFs following knockdown of CDK1 or CDK2 by siRNAs. (**G**) Immunoblot analysis of SOX2 and *O*-GlcNAc levels in HCT116, SW480, DLD1, H460, and A549 cancer cells treated with dinaciclib (0.1 µM) for 24 h. Data for D and E are presented as the means ± SD of three independent experiments. Statistical analysis was performed with Student’s t-tests. (**A**)–(**C**), (**F**) and (**G**): The band intensity is provided under each band. The intensity in panel (**G**) was compared with vehicle-treated control cells for each cell line. Uncropped blot images are presented in Supplementary Fig. S5.

*O*-GlcNAcylation is the post-translational addition of N-acetylglucosamine (also known as *O*-GlcNAc) to serine or threonine residues of proteins that contributes to stability and activity of the modified protein. *O*-GlcNAcylation is a reversible modification mediated by *O*-GlcNAc transferase (OGT) and *O*-GlcNAcase (OGA); OGT catalyses the addition of *O*-GlcNAc to the target protein, whereas OGA removes *O*-GlcNAc from proteins by hydrolysis^53^. Previously, we reported that enhanced *O*-GlcNAcylation is important for SOX2 expression and maintenance of CSC properties, including sphere- and tumour-forming activities, in colon and lung cancer cells^21^. These findings suggested the possibility that *O*-GlcNAc modifications are involved in acquisition of CSC properties. Therefore, we determined the *O*-GlcNAc levels in *HRAS*^*V12*^-expressing *p53*^−/−^ MEFs and TIG-3–SMR cells and found elevated levels of protein *O*-GlcNAcylation compared with those in the respective control cells (Fig. 5A and B). Next, we analysed the cells after treatment with OSMI1, a cell-permeable, small molecule OGT inhibitor^37^. OSMI1 treatment suppressed total *O*-GlcNAcylation levels of proteins and SOX2 expression in these cells (Fig. 5A and B). In contrast, treatment of *HRAS*^*V12*^-expressing *p53*^−/−^ MEFs with thiamet G, a specific OGA inhibitor that increases *O*-GlcNAcylation^38^, enhanced total *O*-GlcNAcylation levels and SOX2 expression (Fig. 5C). Interestingly, the OSMI1-mediated reduction in SOX2 levels was attenuated by treatment with the proteasome inhibitor MG-132 (Fig. 5A and B). The mRNA levels of *SOX2* in *HRAS*^*V12*^-expressing *p53*^*−/−*^ MEFs were not significantly changed by OSMI1 and thiamet G (Fig. 5D), indicating that *O*-GlcNAcylation regulated SOX2 expression at the post-transcriptional level. Consistent with these results, the numbers of sphere-forming cells decreased and increased after treatment with OSMI1 and thiamet G, respectively (Fig. 5E). These results suggest that increased *O*-GlcNAcylation is required for SOX2 protein expression and sphere-forming activity in these cells.

Finally, we analysed the role of RAS/MAPK-activated CDK1 in the induction of *O*-GlcNAcylation. The elevated levels of *O*-GlcNAcylation in *HRAS*^*V12*^-expressing *p53*^*−/−*^ MEFs and TIG-3–SMR cells were suppressed by dinaciclib in a dose dependent manner (Supplementary Fig. S4A and B online). The MEK inhibitors U0126 and PD184352 reduced the phosphorylation of CDK1^T161^ and *O*-GlcNAcylation levels in *HRAS*^*V12*^-expressing *p53*^*−/−*^ MEFs (Supplementary Fig. S4C online). Furthermore, knockdown of CDK1, but not CDK2, with siRNA inhibited *O*-GlcNAcylation levels in these cells (Fig. 5F). In contrast, treatment with palbociclib had no effect on *O*-GlcNAcylation levels (Supplementary Fig. S3B and C online). In addition, *SOX2* expression (as shown in Fig. 4F and G) and *O*-GlcNAcylation levels in KRAS-activated cancer cells were suppressed by dinaciclib (Fig. 5G). These results suggest that RAS/RAF/MAPK pathway-induced CDK1 activation is important for induction of *O*-GlcNAcylation, and this activation pathway is required for SOX2 expression and subsequent CSC generation.

## Discussion

Accumulating evidence has revealed that serial oncogenic mutations in stem cells and even in differentiated somatic cells may induce the generation of CSCs^54–58^. The dedifferentiation of somatic cells into CSCs is thought to be induced by a reprogramming mechanism similar to that observed in the production of iPSCs^59,60^. Viewed through the “hallmarks of cancer”^7,8^, CSCs may be generated by iPSC reprogramming factors that are induced by gain-of-function oncogenic and loss-of-function tumour suppressor gene mutations. Therefore, the *HRAS*^*V12*^-expressing *p53*^*−/−*^ MEFs provided a simple and easy-to-analyse model for evaluating the signalling system used by oncogenes to regulate reprogramming factors. In our previous studies, we found that the *HRAS*^*V12*^ mutation conferred tumour-initiating activity, a hallmark of CSCs, in *p53*^*−/−*^ MEFs^26^, and this phenomenon was completely dependent on NF-κB-induced aerobic glycolysis^13^. In this study, we found that *HRAS*^*V12*^ induced MAPK-CDK1 signal-dependent induction of *SOX2* mRNA transcription and *O*-GlcNAcylation-mediated SOX2 protein accumulation. Although the transcriptional induction mechanism of *SOX2* by CDK1 was not elucidated in this experimental system, CDK1-mediated induction of *SOX2* has been analysed in other studies. For example, it was reported that CDK1-induced phosphorylation directly activated transcription factor CP2-like protein 1 (TFCP2L1), which is an activator of pluripotency-associated genes, including *SOX2*^61^. CDK1 phosphorylated and inhibited the histone lysine demethylase KDM5B, which is a transcriptional suppressor of the pluripotency genes *SOX2* and *NANOG*^62^.

It has been shown that the hexosamine biosynthetic pathway shunts glycolysis toward the production of a key substrate for *O*-GlcNAcylation and is activated by enhanced glycolysis^63^. The RAS/MAPK pathway induces metabolic reprogramming, including enhanced glycolysis^64^. Therefore, in addition to NF-κB-induced enhancement of glycolysis in *p53*^*−/−*^ MEFs^13^, our present results suggest that the RAS/MAPK/CDK1 pathway further promoted glycolysis and resulted in enhanced protein *O*-GlcNAcylation. Although SOX2 expression was induced at the mRNA level in transformed cells (Fig. 1E), we also found that the *O*-GlcNAc modification regulated SOX2 at the post-transcriptional level (Fig. 5A–D). For this issue, we demonstrated that depletion of the *SOX2* gene in *HRAS*^*V12*^-expressing *p53*^*−/−*^ MEFs reduced *O*-GlcNAc levels (Supplementary Fig. S4D online), and exogenous SOX2 expression induced protein *O*-GlcNAcylation in *p53*^*−/−*^ MEFs (Supplementary Fig. S4E online). These data suggest that transcriptionally elevated SOX2 expression promotes *O*-GlcNAc levels, and enhanced *O*-GlcNAcylation could promote expression of SOX2 at post-transcriptional levels. At present, the mechanism by which SOX2 protein expression is induced by *O*-GlcNAcylation has not been elucidated. In this study, we found that *O*-GlcNAcylation inhibited proteasomal degradation of SOX2. Moreover, the *O*-GlcNAc modification of SOX2 was confirmed in *HRAS*^*V12*^-expressing *p53*^*−/−*^ MEFs (Supplementary Fig. S4F online). However, because the band intensities were relatively low, it was difficult to conclude that only direct *O*-GlcNAcylation mediated SOX2 protein induction. These results suggested that direct *O*-GlcNAcylation of SOX2 and/or other factor(s) is involved in the ubiquitin-mediated degradation system that regulates SOX2 protein induction.

The tumour suppressor protein p53 is referred to as "the guardian of the genome" because it continuously surveys damaged genomic DNA and facilitates DNA repair^65^. Moreover, in response to oncogenic signalling, the p53 tumour surveillance system eliminates cells through p53-mediated apoptosis or induction of senescence^66^. In addition to these functions, accumulating evidence has indicated that regulation of cellular metabolism by p53 is involved in tumour suppression^67^. It was demonstrated that mice expressing acetylation-defective p53 mutants lost the ability to induce apoptosis and senescence but retained tumour suppressive function and the ability to modulate the expression of metabolic genes^68^. In *p53*^*−/−*^ MEFs, we previously found that oncogenic transformation by *HRAS*^*V12*^ was dependent on enhanced aerobic glycolysis through NF-κB-mediated induction of the glucose transporter GLUT3^13^. In this previous study, we found that *GLUT3* (also known as *SLC2A3*) functioned as an oncogene because *p53*^*−/−*^ MEFs underwent oncogenic transformation after overexpression of *GLUT3* without *HRAS*^*V12*^. These results suggested that enforced glucose flux induced CSC generation in cells that lacked p53 function. Furthermore, we found that *O*-GlcNAcylation increased with accelerated glucose consumption in *p53*^*−/−*^ MEFs and was further enhanced by *HRAS*^*V12*^ expression^14^. In the present study, CSC generation in *HRAS*^*V12*^-expressing *p53*^*−/−*^ MEFs was completely dependent on the induction of SOX2, and enhancement of *O*-GlcNAcylation by the HRAS^V12^-MAPK-CDK1 pathway was important for SOX2 induction. Therefore, it is possible that glycolysis may be hampered by p53, which prevents excessive *O*-GlcNAcylation-mediated induction of SOX2. Indeed, it has been shown that oncogenes, such as *HRAS*^*V12*^, activate p53 in untransformed cells^66^, and activation of p53 results in inhibition of glucose consumption and glycolysis^13,69^. Moreover, it has been reported that the OKSM reprogramming factors induce p53-mediated senescence^70^, suggesting the possibility that these reprogramming factors limit their own function using p53-mediated negative feedback. Therefore, at least in MEFs, p53 may exert a tumour suppressor function through the regulation of *O*-GlcNAcylation by limiting glucose metabolism.

In MEFs, induction of *SOX2* was essential for CSC reprogramming by *HRAS*^*V12*^, but this pathway may not be the only signal for CSC generation. For example, in the context of inflammation-induced cancer, we found that the activated form of MYD88, a regulator of inflammatory signalling, promoted CSC generation in *p53*^*−/−*^ MEFs through activation of the NF-κB-HIF1-OCT4, but not the SOX2, induction pathway^71^. Furthermore, in the present study, expression of *HRAS*^*V12*^ alone was insufficient to induce *SOX2* in human TIG-3 cells. Although it is possible that this difference was because of cell type specificity, differences between human and rodent cells cannot be ruled out. In contrast to human cells, primary rodent cells are efficiently converted to tumorigenic cells by the co-expression of oncogenes, suggesting a fundamental difference between humans and rodents^32^. Therefore, these results suggest the possibility that an unidentified tumour suppressive mechanism other than p53 exists in humans and prevents the induction of SOX2 by RAS. Further analyses are required to clarify this mechanism.

## Materials and methods

### Cell culture and reagents

The *p53*^*−/−*^MEFs, *HRAS*^*V12*^-expressing *p53*^*−/−*^MEFs, TIG-3, and TIG-3–SMR cells were prepared as previously described^13,14^. The *HRAS*^*V12*^ mutant was based on the National Center for Biotechnology Information (NCBI, USA) reference sequence NM_001130442.3. The NCBI reference sequences for SV40 T antigen and *c-MYC* were NC_001669.1 and NM_001354870.1, respectively. These cells were cultured in DMEM (Nissui, Japan) containing 10% foetal bovine serum (FBS). HCT116, SW480, and DLD1 colon cancer cell lines and HCC827 and H460 lung cancer cell lines were purchased from ATCC (Manassas, VA, USA). HCT116 cells were cultured in McCoy's 5A medium (Gibco, Waltham, MA, USA), SW480 and DLD1 cells were grown in DMEM, and HCC827 and H460 cells were cultured in RPMI 1640 medium (Nissui, Japan), each containing 10% FBS. All cells were tested for mycoplasma contamination using the MycoAlert™ Mycoplasma Detection Kit (Lonza, Switzerland), which confirmed the absence of mycoplasma. U0126 (Promega, Madison, WI, USA), LY294002 (Fujifilm Wako, Japan), PD184352, dinaciclib, palbociclib (Selleck Biotech, Houston, TX, USA), thiamet G (Sigma-Aldrich, St. Louis, MO, USA), and OSMI1 (Cayman Chemical, Ann Arbor, MI, USA) were used in the inhibition experiments. MG-132, and anti-c-H-Ras (OP23) and anti-SV40 T antigen (DP01) antibodies were obtained from Calbiochem (Merck, Germany). Anti-OGT (H-300), anti-TERT (H-231), and anti-c-Myc (9E10) antibodies were obtained from Santa Cruz Biotechnology (Dallas, TX, USA). Anti-*O*-GlcNAc antibody (CTD110.6) was purchased from Covance (Princeton, NJ, USA). Anti-SOX2 (D9B8N), anti-ERK1/2 (#9102), and anti-pERK1/2^T202/Y204^ (#9101) antibodies were purchased from Cell Signaling Technology (Danvers, MA, USA). Anti-CDK1 (A2861), anti-pCDK1^T161^ (AP0324), and anti-CDK2 (A0294) antibodies were obtained from ABclonal (Cambridge, MA, USA). Anti-β-actin (AC-74) was obtained from Sigma-Aldrich. All antibodies were used at a 1:1000 dilution.

### Expression vectors and virus infection

A SOX2 sgRNA CRISPR/Cas9 All-in-One Lentivirus vector set (Applied Biological Materials, Canada) was used for knockout of the *SOX2* gene. The sgRNA sequences were as follows: sgRNA#1, CAACCAGAAGAACAGCC; sgRNA#2, TCATCGACGAGGCCAAG; and sgRNA#3, ATTATAAATACCGGCCG. For knockout of the *p53* gene in TIG-3 cells, the lentivirus vector pSpCas9(BB)-2A-Puro (PX459) V2.0 was obtained from the Addgene repository (plasmid #62988, Cambridge, MA, USA), and the target sequence for *p53* (ACCAGCAGCTCCTACACCGG) was cloned into the PX459 vector following the repository’s recommended Zhang lab protocol. Retroviral vectors pMXs-mSOX2-IP (plasmid #15919), pBabe BRAF^V600E^ (plasmid #17544), and pBabe PI3K p110^CAAX^ (plasmid #13339) containing the puromycin resistance marker were purchased from Addgene. The pBabe SV40 T antigen, pBabe c-Myc and pBabe HRAS^V12^ vectors were used with blasticidin, neomycin, and hygromycin selectable markers, respectively. Lentiviral and retroviral infections were performed as previously described^23,64^. The infected cells were selected with the appropriate antibiotics.

### siRNA and transfection

The predesigned short interfering RNA (siRNA) for mouse *Cdk1* and *Cdk2* were obtained from Sigma-Aldrich: siCDK1#1, SASI_Mm01_00179321; siCDK1#2, SASI_Mm01_00179322; siCDK2#1, SASI_Mm02_00323492; siCDK2#2, SASI_Mm01_00151932. We performed reverse transfection of siRNA using Lipofectamine™ RNAiMAX Transfection Reagent (Thermo Fisher Scientific, Waltham, MA, USA).

### Immunoprecipitation and immunoblot analysis

Cells were lysed with TNE buffer (10 mM Tris–HCl, pH 7.4; 1% NP-40; 150 mM NaCl; 1 mM EDTA; 1 mM DTT, and protease inhibitor cocktail) (Nacalai Tesque, Japan). Protein concentrations were measured using the Bradford assay. For immunoprecipitation, lysates were pre-cleared with Protein A/G PLUS agarose (Santa Cruz Biotechnology), and proteins were immunoprecipitated with the anti-SOX2 antibody. For immunoblot analysis, cellular proteins (20µ) or immunoprecipitates were separated using sodium dodecyl sulfate–polyacrylamide gel electrophoresis and then transferred to polyvinylidene difluoride membranes (Merck). The membranes were probed with primary antibodies, followed by incubation with horseradish peroxidase-conjugated mouse or rabbit immunoglobulin G (GE Healthcare, England) and visualisation using Chemi-Lumi-One Super or Ultra assay kits (Nacalai Tesque). The protein bands were digitalised using the LAS-3000 mini image analyser (Fujifilm, Japan), and the intensity of each band was quantified using ImageJ software. For quantification, the intensity of the β-actin band was used to normalise each protein signal.

### Quantitative real-time PCR

Total RNA was extracted using the NucleoSpin RNA kit (Macherey–Nagel, Germany) following the manufacturer’s instructions. Double-stranded cDNA was prepared from total RNA using oligonucleotide (dT), random primers, and Superscript III reverse transcriptase (Invitrogen, Carlsbad, CA, USA). Quantitative real-time PCR (qPCR) analysis was performed as previously described^23^. The following probes were predesigned from Applied Biosystems® (Thermo Fisher Scientific): mouse β-actin, Mm00607939_s1; mouse *Oct4*, Mm03053917_g1; mouse *Klf4*, Mm00516104_m1; mouse *Sox2*, Mm03053810_s1; human β-actin, Hs00357333_g1; human *OCT4*, Hs03005111_m1; human *KLF4*, Hs00358836_m1; human *SOX2*, Hs01053049_s1; and human *NANOG*, Hs04260366_m1.

### Immunofluorescent analysis

The *p53*^−/−^ MEFs and *HRAS*^*V12*^-expressing *p53*^−/−^ MEFs (4 × 10^4^) were cultured on glass coverslips in 6-well plates and treated with U0126 (10 µM) or LY294002 (20 µM) for 24 h. The cells were washed in phosphate-buffered saline (PBS), fixed with 4% paraformaldehyde in PBS for 15 min, and permeabilised with 0.5% Triton X-100 in PBS for 10 min. Permeabilised cells were blocked with Blocking One Histo reagent (Nacalai Tesque) for 30 min and incubated with anti-SOX2 antibody for 1 h at room temperature. Alexa Flour 488-conjugated anti-rabbit antibody (Thermo Fisher Scientific) was used as the secondary antibody for 1 h at room temperature. VECTASHIELD Mounting Medium with DAPI (Vector Laboratories, Burlingame, CA, USA) was used to stain nuclei and for mounting the cells on slides. Images were acquired using a fluorescence microscope (BioZero BZ-8100; Keyence, Japan), and the fluorescence intensities were quantified with ImageJ software.

### Cell growth analysis

*HRAS*^*V12*^-expressing *p53*^−/−^ MEFs (1 × 10^5^) expressing each *SOX2* sgRNA separately were seeded in 6-well plates. Cell numbers were determined using a Vi-CELL cell analyser (Beckman, Brea, CA, USA) on the indicated days after plating, and cell growth curves were created.

### Sphere formation assay

Cells (5 × 10^3^ or 1 × 10^4^) were plated in 6-well, ultra-low attachment plates and grown in serum-free DMEM/F12 medium containing epidermal growth factor (20 ng/mL) and basic fibroblast growth factor (10 ng/mL) for 7 days. The numbers of spheres were counted in each treatment group. Sphere images were captured using the BZ-8100 microscope.

### Colony formation assay

A layer of 1.5% (weight/volume) agarose prepared in DMEM containing 10% FBS was added to the wells of 6-well plates. Agarose (0.6%) containing 3 × 10^4^ *HRAS*^*V12*^-expressing *p53*^−/−^ MEFs containing each *SOX2* sgRNA was added to the top of the first layer. After 30 days, each well was stained with 0.005% crystal violet (Sigma-Aldrich), and the colonies were counted.

### Animal experiments and cell line xenografts

The animal experiment protocol was approved by the Ethics Committee on Animal Experiments of Nippon Medical School (ethics approval number 26–020, 27–188). Animal experiments were carried out in accordance with the guidelines for Animal Experiments of Nippon Medical School and the guidelines of The Law and Notification of the Government of Japan as well as the ARRIVE guidelines. Mice were maintained at 20–24 °C in a facility with a 12 h light/ dark cycle and 40%–70% humidity. The mice were allowed free access to water and standard MF laboratory mouse chow (Oriental Yeast Co., ltd. Tokyo, Japan) and housed at a maximum number of five per cage. All mice were checked for stress each day. For the xenograft experiments, 5-week-old male BALB/cAJcl-nu/nu mice were purchased from CLEA Japan, Inc. (Tokyo, Japan) and assigned at random to the experiments. These mice were subcutaneously injected with 1 × 10^5^ cells of *p53*^*−/−*^ MEFs, *HRAS*^*V12*^-expressing *p53*^−/−^ MEFs or *HRAS*^*V12*^-expressing *p53*^−/−^ MEFs containing *SOX2* gRNA. The number of mice used are indicated for each experiment. Tumour growth was monitored every 3 days for 3 weeks and each week thereafter by caliper measurements, and tumour size was determined using the following formula: (Length × Width^2^)/2. At the end of the experiments (3 or 13 weeks post-injection), mice were euthanized by cervical dislocation, then each tumour were removed and weighed, and also collected for further experiments.

### Statistical analysis

All experiments were repeated at least three times independently. Data are presented as means ± standard deviation (SD). Statistical analysis was performed with the Student’s *t*-test using Microsoft Excel (Microsoft, DC, USA). *P* < 0.05 was considered statistically significant.

## Supplementary Information

Supplementary Information.

## Data Availability

The data are available from the corresponding author upon request.

## References

[1] Bjerkvig R, Tysnes BB, Aboody KS, Najbauer J, Terzis AJ (2005). Opinion: the origin of the cancer stem cell: current controversies and new insights. Nat. Rev. Cancer.

[2] Batlle E, Clevers H (2017). Cancer stem cells revisited. Nat. Med..

[3] Lytle NK, Barber AG, Reya T (2018). Stem cell fate in cancer growth, progression and therapy resistance. Nat. Rev. Cancer.

[4] Goding CR, Pei D, Lu X (2014). Cancer: pathological nuclear reprogramming?. Nat. Rev. Cancer.

[5] Flavahan WA, Gaskell E, Bernstein BE (2017). Epigenetic plasticity and the hallmarks of cancer. Science.

[6] Ohnishi K (2014). Premature termination of reprogramming in vivo leads to cancer development through altered epigenetic regulation. Cell.

[7] Hanahan D, Weinberg RA (2000). The hallmarks of cancer. Cell.

[8] Hanahan D, Weinberg RA (2011). Hallmarks of cancer: the next generation. Cell.

[9] Vogelstein B, Lane D, Levine AJ (2000). Surfing the p53 network. Nature.

[10] Labuschagne CF, Zani F, Vousden KH (1870). Control of metabolism by p53 - Cancer and beyond. Biochim. Biophys. Acta Rev. Cancer.

[11] Levine AJ (2020). p53: 800 million years of evolution and 40 years of discovery. Nat. Rev. Cancer.

[12] Koppenol WH, Bounds PL, Dang CV (2011). Otto Warburg's contributions to current concepts of cancer metabolism. Nat. Rev. Cancer.

[13] Kawauchi K, Araki K, Tobiume K, Tanaka N (2008). p53 regulates glucose metabolism through an IKK-NF-kappaB pathway and inhibits cell transformation. Nat. Cell Biol..

[14] Kawauchi K, Araki K, Tobiume K, Tanaka N (2009). Loss of p53 enhances catalytic activity of IKKbeta through O-linked beta-N-acetyl glucosamine modification. Proc. Natl. Acad. Sci. U S A.

[15] Hong H (2009). Suppression of induced pluripotent stem cell generation by the p53–p21 pathway. Nature.

[16] Li H (2009). The Ink4/Arf locus is a barrier for iPS cell reprogramming. Nature.

[17] Marion RM (2009). A p53-mediated DNA damage response limits reprogramming to ensure iPS cell genomic integrity. Nature.

[18] Utikal J (2009). Immortalization eliminates a roadblock during cellular reprogramming into iPS cells. Nature.

[19] Tsogtbaatar E, Landin C, Minter-Dykhouse K, Folmes CDL (2020). Energy metabolism regulates stem cell pluripotency. Front. Cell Dev. Biol..

[20] Jang H (2012). O-GlcNAc regulates pluripotency and reprogramming by directly acting on core components of the pluripotency network. Cell Stem Cell.

[21] Shimizu M, Tanaka N (2019). IL-8-induced O-GlcNAc modification via GLUT3 and GFAT regulates cancer stem cell-like properties in colon and lung cancer cells. Oncogene.

[22] Prior IA, Lewis PD, Mattos C (2012). A comprehensive survey of Ras mutations in cancer. Cancer Res..

[23] Pylayeva-Gupta Y, Grabocka E, Bar-Sagi D (2011). RAS oncogenes: weaving a tumorigenic web. Nat. Rev. Cancer.

[24] Simanshu DK, Nissley DV, McCormick F (2017). RAS proteins and their regulators in human disease. Cell.

[25] Lavoie H, Gagnon J, Therrien M (2020). ERK signalling: a master regulator of cell behaviour, life and fate. Nat. Rev. Mol. Cell Biol..

[26] Tanaka N (1994). Cellular commitment to oncogene-induced transformation or apoptosis is dependent on the transcription factor IRF-1. Cell.

[27] Eramo A (2008). Identification and expansion of the tumorigenic lung cancer stem cell population. Cell Death Differ..

[28] Vermeulen L (2008). Single-cell cloning of colon cancer stem cells reveals a multi-lineage differentiation capacity. Proc. Natl. Acad. Sci. U S A.

[29] Bareiss PM (2013). SOX2 expression associates with stem cell state in human ovarian carcinoma. Cancer Res..

[30] Qi XT (2019). KLF4 functions as an oncogene in promoting cancer stem cell-like characteristics in osteosarcoma cells. Acta Pharmacol. Sin..

[31] Wang G, Zhou H, Gu Z, Gao Q, Shen G (2018). Oct4 promotes cancer cell proliferation and migration and leads to poor prognosis associated with the survivin/STAT3 pathway in hepatocellular carcinoma. Oncol. Rep..

[32] Hahn WC (1999). Creation of human tumour cells with defined genetic elements. Nature.

[33] Boehm JS, Hession MT, Bulmer SE, Hahn WC (2005). Transformation of human and murine fibroblasts without viral oncoproteins. Mol. Cell Biol..

[34] Hahn WC (2002). Enumeration of the simian virus 40 early region elements necessary for human cell transformation. Mol. Cell Biol..

[35] Xiang R (2011). Downregulation of transcription factor SOX2 in cancer stem cells suppresses growth and metastasis of lung cancer. Br. J. Cancer.

[36] Zheng J (2017). Sox2 modulates motility and enhances progression of colorectal cancer via the Rho-ROCK signaling pathway. Oncotarget.

[37] Liu P (2018). SOX2 promotes cell proliferation and metastasis in triple negative breast cancer. Front. Pharmacol..

[38] Mori S (2009). Anchorage-independent cell growth signature identifies tumors with metastatic potential. Oncogene.

[39] Downward J (2008). Targeting RAS and PI3K in lung cancer. Nat. Med..

[40] Sherr CJ, Beach D, Shapiro GI (2016). Targeting CDK4 and CDK6: from discovery to therapy. Cancer Discov..

[41] Bustos MA (2017). MiR-200a regulates CDK4/6 inhibitor effect by targeting CDK6 in metastatic melanoma. J. Invest. Dermatol..

[42] Inoue K, Fry EA (2015). Aberrant expression of cyclin D1 in cancer. Sign Transduct Insights.

[43] Fry DW (2004). Specific inhibition of cyclin-dependent kinase 4/6 by PD 0332991 and associated antitumor activity in human tumor xenografts. Mol. Cancer Ther..

[44] Borysov SI, Guadagno TM (2008). A novel role for Cdk1/cyclin B in regulating B-raf activation at mitosis. Mol. Biol. Cell.

[45] Keenan SM, Bellone C, Baldassare JJ (2001). Cyclin-dependent kinase 2 nucleocytoplasmic translocation is regulated by extracellular regulated kinase. J. Biol. Chem..

[46] Lunn CL, Chrivia JC, Baldassare JJ (2010). Activation of Cdk2/Cyclin E complexes is dependent on the origin of replication licensing factor Cdc6 in mammalian cells. Cell Cycle.

[47] Duong MT (2013). Hbo1 is a cyclin E/CDK2 substrate that enriches breast cancer stem-like cells. Cancer Res..

[48] Neganova I (2014). CDK1 plays an important role in the maintenance of pluripotency and genomic stability in human pluripotent stem cells. Cell Death Dis.

[49] Peyressatre M, Prevel C, Pellerano M, Morris MC (2015). Targeting cyclin-dependent kinases in human cancers: from small molecules to Peptide inhibitors. Cancers (Basel).

[50] Parry D (2010). Dinaciclib (SCH 727965), a novel and potent cyclin-dependent kinase inhibitor. Mol. Cancer Ther..

[51] Meijer L (1997). Biochemical and cellular effects of roscovitine, a potent and selective inhibitor of the cyclin-dependent kinases cdc2, cdk2 and cdk5. Eur. J. Biochem..

[52] Solomon MJ, Lee T, Kirschner MW (1992). Role of phosphorylation in p34cdc2 activation: identification of an activating kinase. Mol. Biol. Cell.

[53] Yuzwa SA (2008). A potent mechanism-inspired O-GlcNAcase inhibitor that blocks phosphorylation of tau in vivo. Nat. Chem. Biol..

[54] Afify SM, Seno M (2019). Conversion of stem cells to cancer stem cells: undercurrent of cancer initiation. Cancers (Basel).

[55] Ayob AZ, Ramasamy TS (2018). Cancer stem cells as key drivers of tumour progression. J. Biomed. Sci..

[56] Jin X, Jin X, Kim H (2017). Cancer stem cells and differentiation therapy. Tumour Biol..

[57] Kreso A, Dick JE (2014). Evolution of the cancer stem cell model. Cell Stem Cell.

[58] Plaks V, Kong N, Werb Z (2015). The cancer stem cell niche: how essential is the niche in regulating stemness of tumor cells?. Cell Stem Cell.

[59] Friedmann-Morvinski D, Verma IM (2014). Dedifferentiation and reprogramming: origins of cancer stem cells. EMBO Rep..

[60] Takahashi K, Yamanaka S (2016). A decade of transcription factor-mediated reprogramming to pluripotency. Nat. Rev. Mol. Cell Biol..

[61] Heo J (2020). Phosphorylation of TFCP2L1 by CDK1 is required for stem cell pluripotency and bladder carcinogenesis. EMBO Mol. Med..

[62] Yeh IJ (2019). Phosphorylation of the histone demethylase KDM5B and regulation of the phenotype of triple negative breast cancer. Sci. Rep..

[63] McClain DA, Crook ED (1996). Hexosamines and insulin resistance. Diabetes.

[64] Kimmelman AC (2015). Metabolic dependencies in RAS-driven cancers. Clin. Cancer Res..

[65] Lane DP (1992). Cancer. p53, guardian of the genome. Nature.

[66] Lowe SW, Cepero E, Evan G (2004). Intrinsic tumour suppression. Nature.

[67] Vousden KH, Ryan KM (2009). p53 and metabolism. Nat. Rev. Cancer.

[68] Li T (2012). Tumor suppression in the absence of p53-mediated cell-cycle arrest, apoptosis, and senescence. Cell.

[69] Bensaad K (2006). TIGAR, a p53-inducible regulator of glycolysis and apoptosis. Cell.

[70] Mosteiro L (2016). Tissue damage and senescence provide critical signals for cellular reprogramming in vivo. Science.

[71] Tanimura A, Nakazato A, Tanaka N (2021). MYD88 signals induce tumour-initiating cell generation through the NF-kappaB-HIF-1alpha activation cascade. Sci. Rep..

