# Supplementary material for: Enhanced O-GlcNAc modification induced by the RAS/MAPK/CDK1 pathway is required for SOX2 protein expression and generation of cancer stem cells
Source: Sci Rep. 2022 Feb 21;12:2910. doi: 10.1038/s41598-022-06916-y (PMC8861017; doi:10.1038/s41598-022-06916-y)

**Supplementary Information**

**Title**

Enhanced *O*-GlcNAc modification induced by the RAS/MAPK/CDK1 pathway is required for SOX2 protein expression and generation of cancer stem cells

**Authors**

Masahiro Shimizu, Hiroshi Shibuya, and Nobuyuki Tanaka

**Supplementary Figures 1-5**


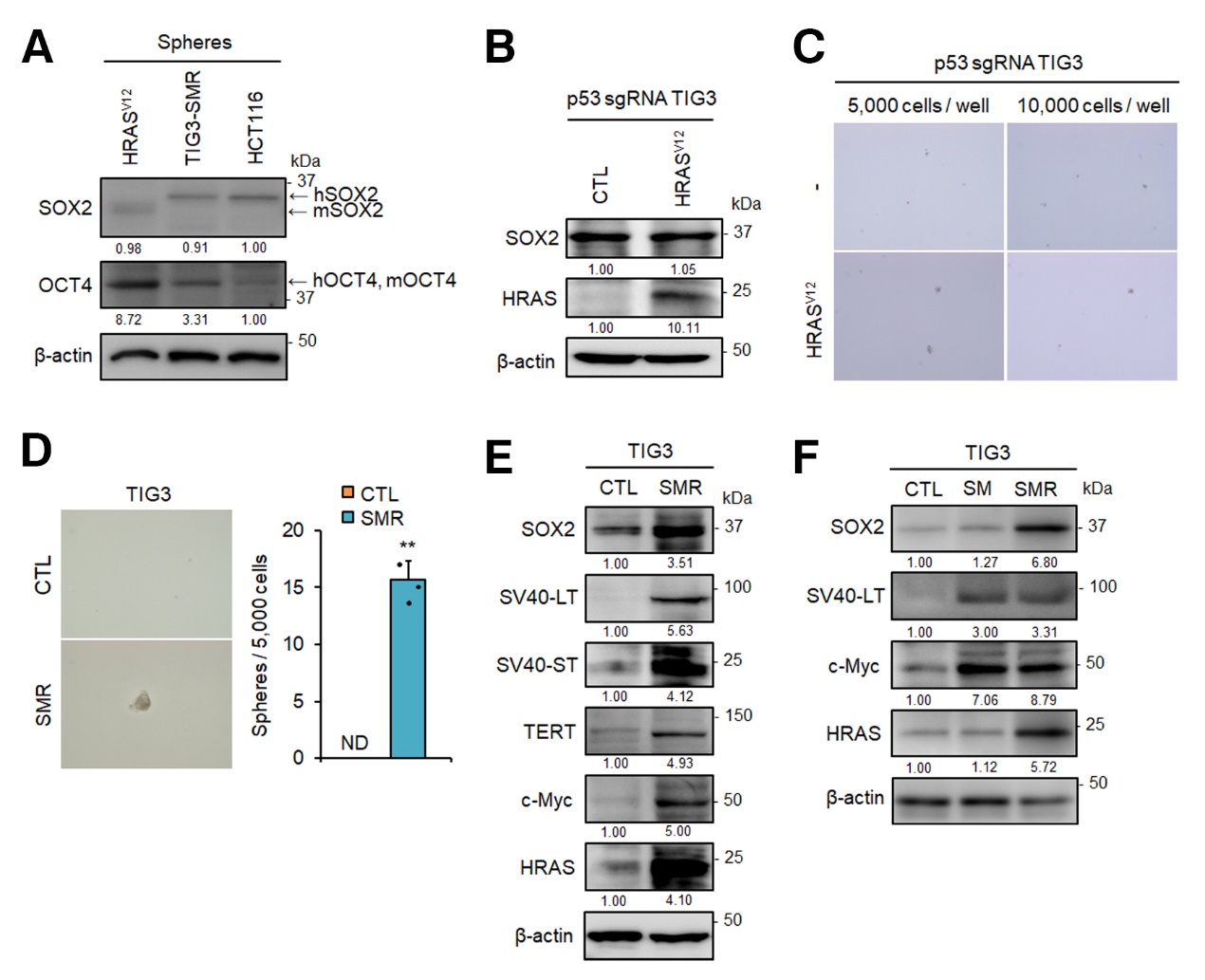


**Supplementary Figure 1.** Oncogenic mutations causing transformation are required for SOX2 expression and sphere formation in TIG-3 cells**. A,** Expression of human SOX2 (hSOX2), mouse SOX2 (mSOX2), human OCT4 (hOCT4), and mouse OCT4 (mOCT4) in sphere-forming cells from *HRAS^V12^*-expressing *p53*^−/−^ mouse embryonic fibroblasts (MEFs), human TIG-3-SMR cells, and the human colon cancer line HCT116. **B**, p53-deficient human TIG-3 cells were generated by co-expressing p53 sgRNA with CRISPR and Cas9 protein using lentiviral infection for 2 days and selection with puromycin for 3 days. The HRAS^V12^ mutant protein was stably expressed in these cells by retroviral infection for 2 days and selection with hygromycin for 3 days. Expression of HRAS and SOX2 was confirmed by immunoblot analysis of the *HRAS^V12^*-expressing *p53* knockout TIG-3 cells. **C**, Sphere formation in *HRAS^V12^*-expressing *p53* knockout TIG-3 cells. Representative images of spheres from 5,000 and 10,000 cells are shown. **D**, SV-40, c-Myc, and HRAS^V12^ were stably expressed in TIG-3 (TIG-3–SMR) cells by retroviral infection for 2 days, and the cells were selected with blasticidin, neomycin, and hygromycin for 3 days. Representative images of sphere formation in TIG-3–SMR cells are shown on the left; the graph on the right shows quantification of the numbers of spheres counted per 5,000 cells. Significance was confirmed by comparisons between the number of spheres in TIG-3 (CTL) and TIG-3–SMR cells. ND, none detected. ***P* < 0.01. **E** and **F,** Western blot analysis of the indicated proteins in TIG-3-SM and TIG-3–SMR cells. A, B, E and F: The band intensity is provided under each band.


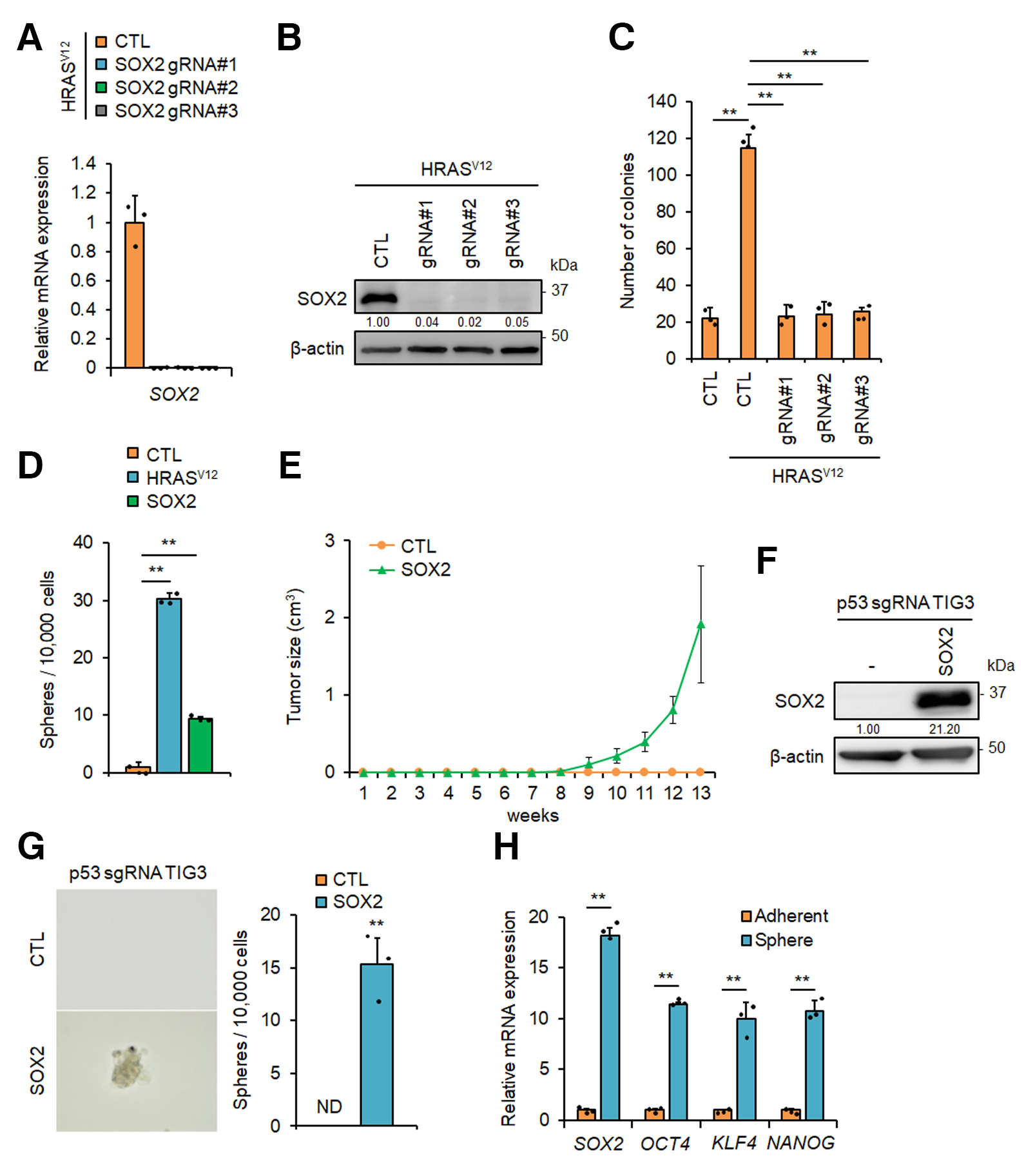


**Supplementary Figure 2.** SOX2 expression is required for colony, sphere, and tumour formation. **A** and **B**, *SOX2* sgRNAs were co-expressed with CRISPR and Cas9 proteins in *HRAS^V12^*-expressing *p53*^−/−^ MEFs. Expression of SOX2 was confirmed by qPCR analysis (A) and immunoblotting (B). **C**, Colony formation assay of *HRAS^V12^*-expressing *p53*^−/−^ MEFs containing each SOX2 sgRNA. ***P* < 0.01. **D**, SOX2 was stably expressed in *p53*^−/−^ MEFs by retroviral infection for 2 days, and cells were selected with puromycin for 3 days. The numbers of spheres formed from *HRAS^V12^*- or *SOX2*-expressing *p53*^−/−^ MEFs were counted after 7 days of culture. Significance was confirmed by comparison with the number of spheres from *p53*^−/−^ MEFs expressing only the vector (CTL). ***P* < 0.01. **E**, *p53*^−/−^ MEFs (CTL) or *SOX2*-expressing *p53*^−/−^ MEFs (1 × 10^6^) were subcutaneously injected into immunodeficient mice (n = 5 per group). Tumour sizes were measured each week. **F**, SOX2 was stably expressed in *p53* knockout TIG-3 cells. SOX2 expression was confirmed by immunoblotting. **G**, Sphere formation of cells indicated in (F) after 7 days of culture. Representative images are shown on the left, and quantification is shown on the right. ND, none detected. ***P* < 0.01. **H**, qPCR analysis of the stem cell marker genes *SOX2*, *OCT4*, *KLF4*, and *NANOG* in adherent and sphere-forming cells indicated in (G). ***P* < 0.01. A, C, D, G and H: Data are presented as the means ± SD of three independent experiments. Statistical analysis was performed with Student’s t-tests. B and F: The band intensity is provided under each bands.


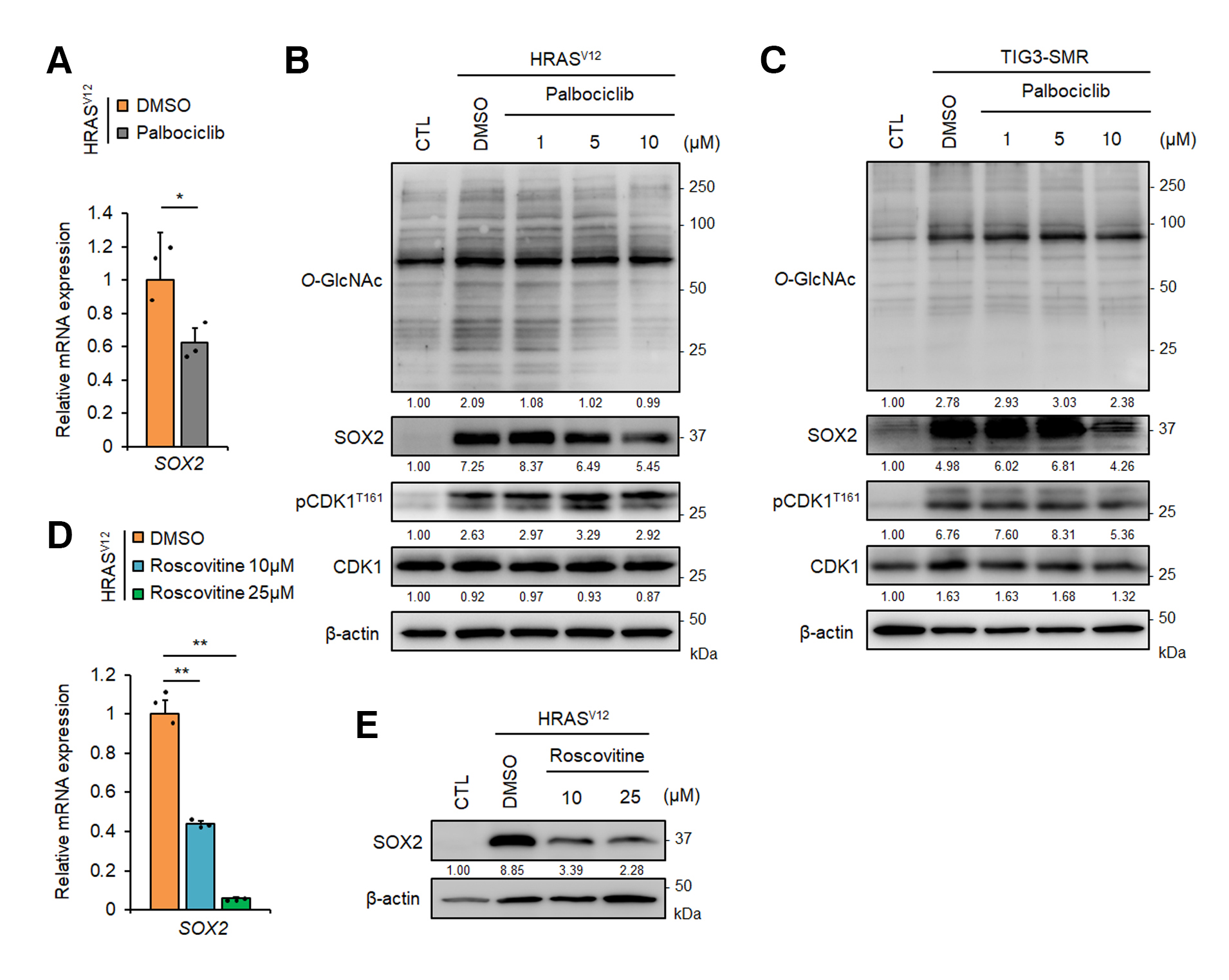


**Supplementary Figure 3.** CDK inhibitors suppress SOX2 expression.**A**, Expression levels of *SOX2* mRNA in *HRAS^V12^*-expressing *p53*^−/−^ mouse embryonic fibroblasts (MEFs) treated with palbociclib (10 µM) for 24 h. **P* < 0.05. **B** and **C**, Immunoblotting analysis of *O*-GlcNAc-modified proteins, SOX2 expression, and active pCDK1 in *HRAS^V12^*-expressing *p53*^−/−^ MEFs (B) and human TIG-3–SMR cells (C) treated with vehicle (DMSO) or palbociclib (1, 5, 10 µM) for 24 h. **D** and **E**, qPCR analysis (D) and immunoblotting (E) of SOX2 in *HRAS^V12^*-expressing *p53*^−/−^ MEFs treated with the CDK inhibitor roscovitine (10, 25 µM) for 24 h. ***P* < 0.01. A and D: Data are presented as the means ± SD of three independent experiments. Statistical analysis was performed with Student’s t-tests. B-C and E: The band intensity is provided under each bands.


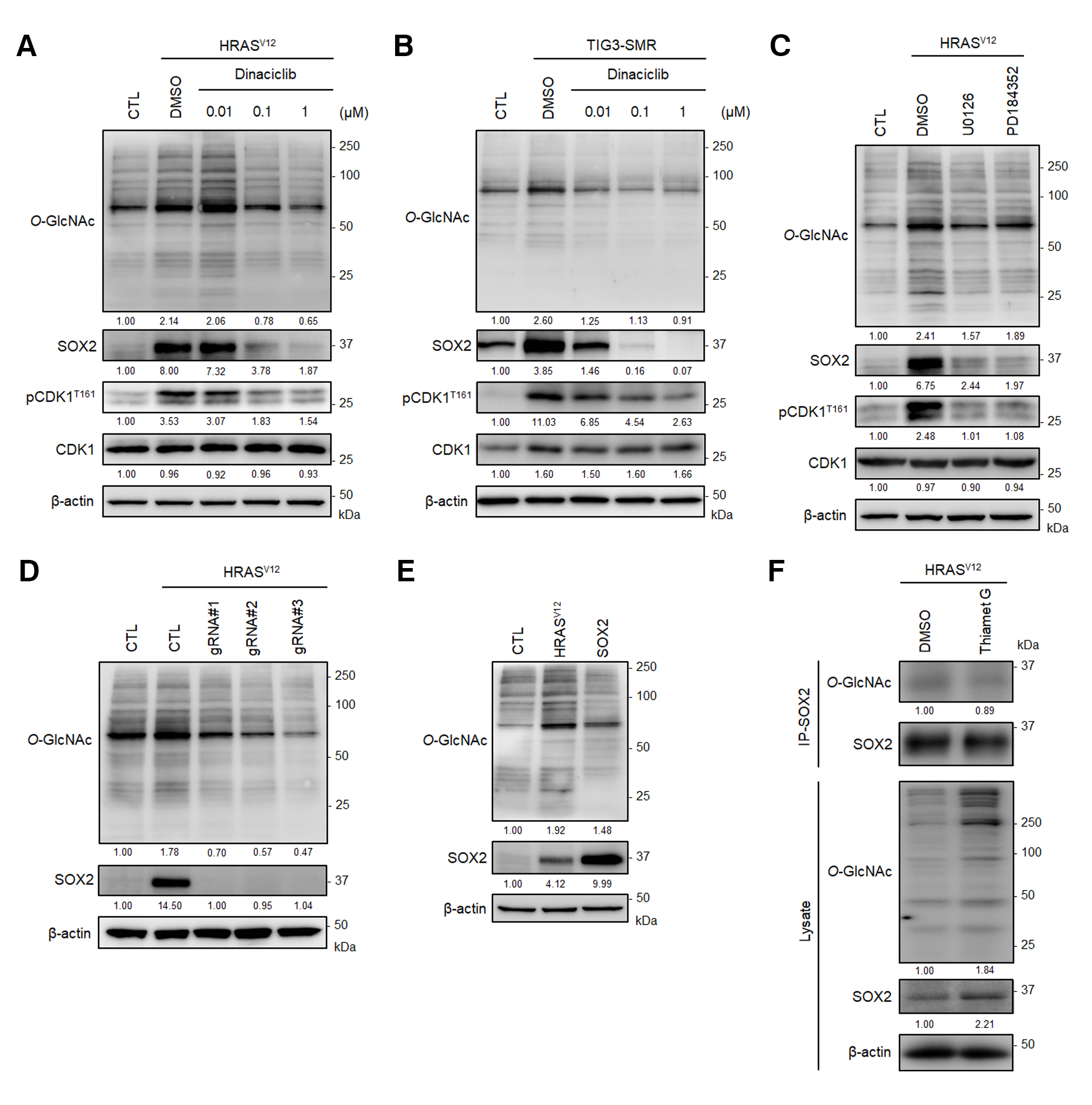


**Supplementary Figure 4.** *O*-GlcNAc levels correlate with SOX2 expression. **A** and **B**, Western blot analysis of *O*-GlcNAc-modified proteins, SOX2 expression, and active pCDK1 in *HRAS^V12^*-expressing *p53*^−/−^ mouse embryonic fibroblasts (MEFs) (A) and human TIG-3–SMR cells (B) treated with vehicle (DMSO) or dinaciclib (0.01, 0.1, 1 µM) for 24 h. **C**, Immunoblotting of *O*-GlcNAc-modified proteins, SOX2, and pCDK1 in *HRAS^V12^*-expressing *p53*^−/−^ MEFs treated with vehicle, U0126 (10 µM), or PD184352 (1 µM) for 24 h. **D**, Immunoblotting assay of *O*-GlcNAcylation levels in *HRAS^V12^*-expressing *p53*^−/−^ MEFs containing each *SOX2* sgRNA. **E**, Expression levels of *O*-GlcNAc-modified proteins in *HRAS^V12^*-expressing *p53*^−/−^ MEFs and *SOX2*-expressing *p53*^−/−^ MEFs. **F**, Immunoprecipitation and immunoblot analysis of *O*-GlcNAc modification on SOX2 in HRAS^V12^-expressing p53^−/−^ MEFs treated with vehicle or thiamet G (10µM) for 24h. A-F: The band intensity is provided under each bands.

**Supplementary Figure 5.** The original images in all Figures and Supplementary Figures. Some membranes were cut prior to hybridization with antibodies.


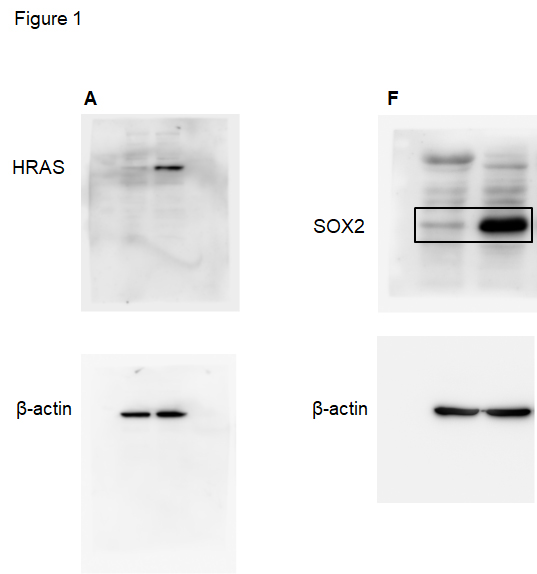


**Supplementary Figure 5 continued**


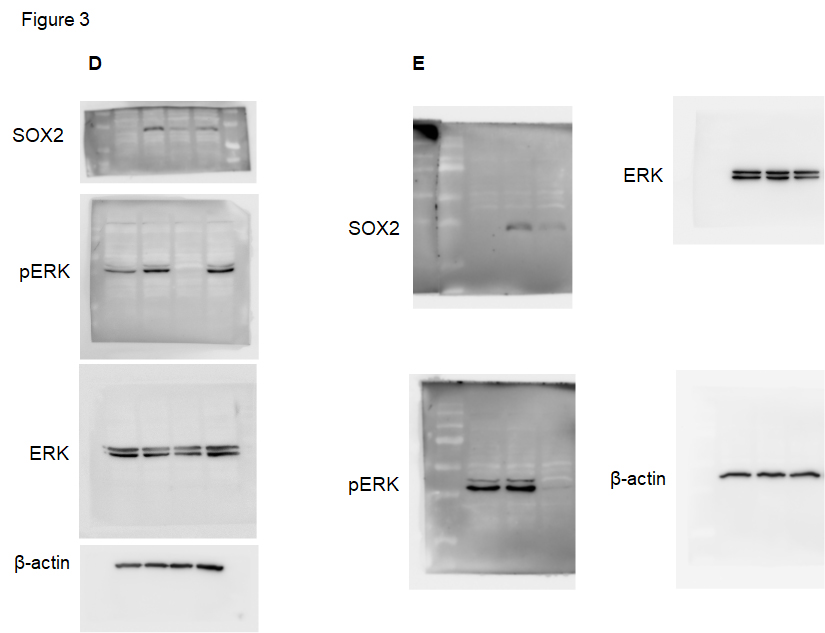


**Supplementary Figure 5 continued**


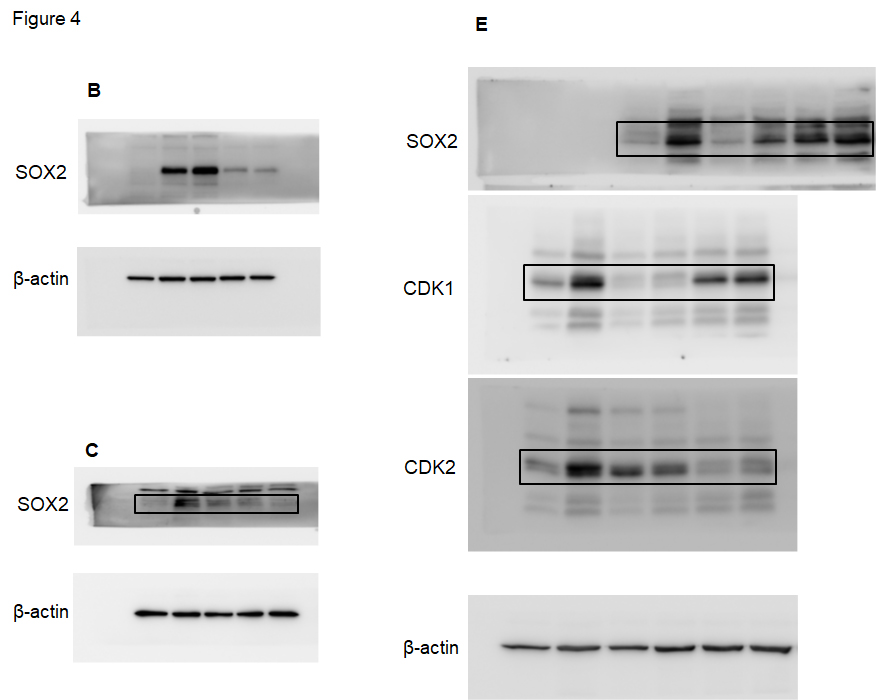


**Supplementary Figure 5 continued**


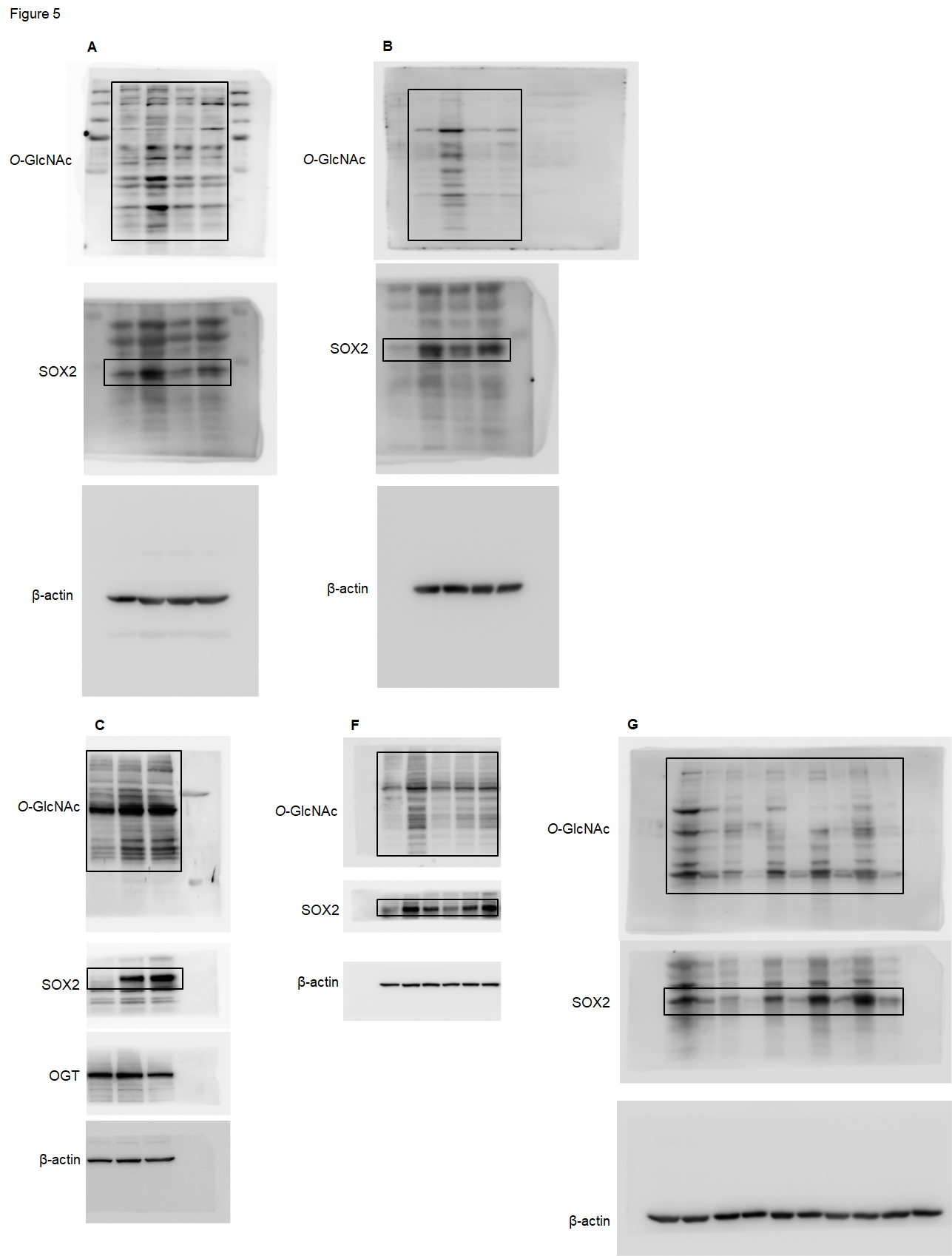


**Supplementary Figure 5 continued**


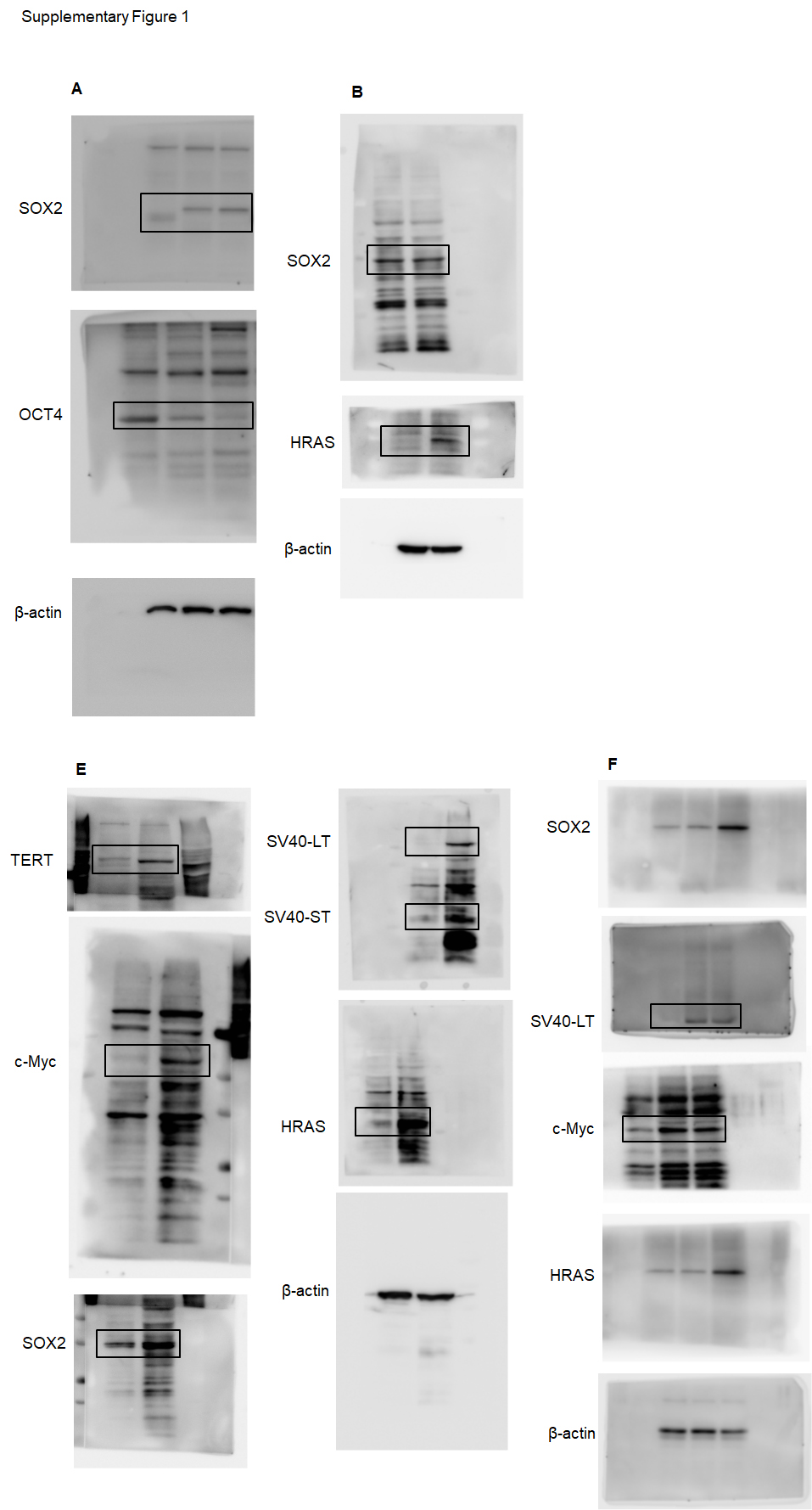


**Supplementary Figure 5 continued**


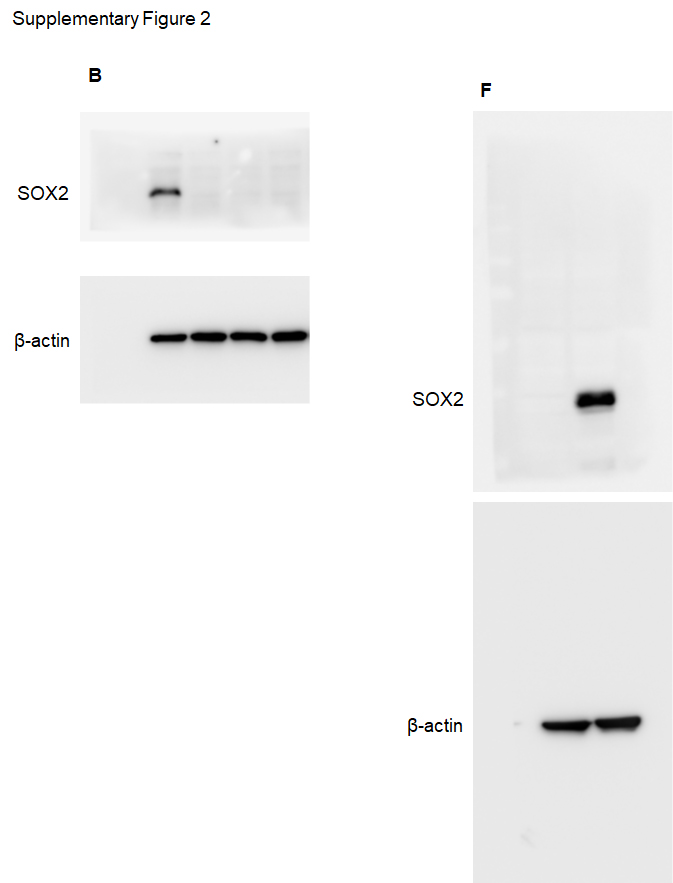


**Supplementary Figure 5 continued**


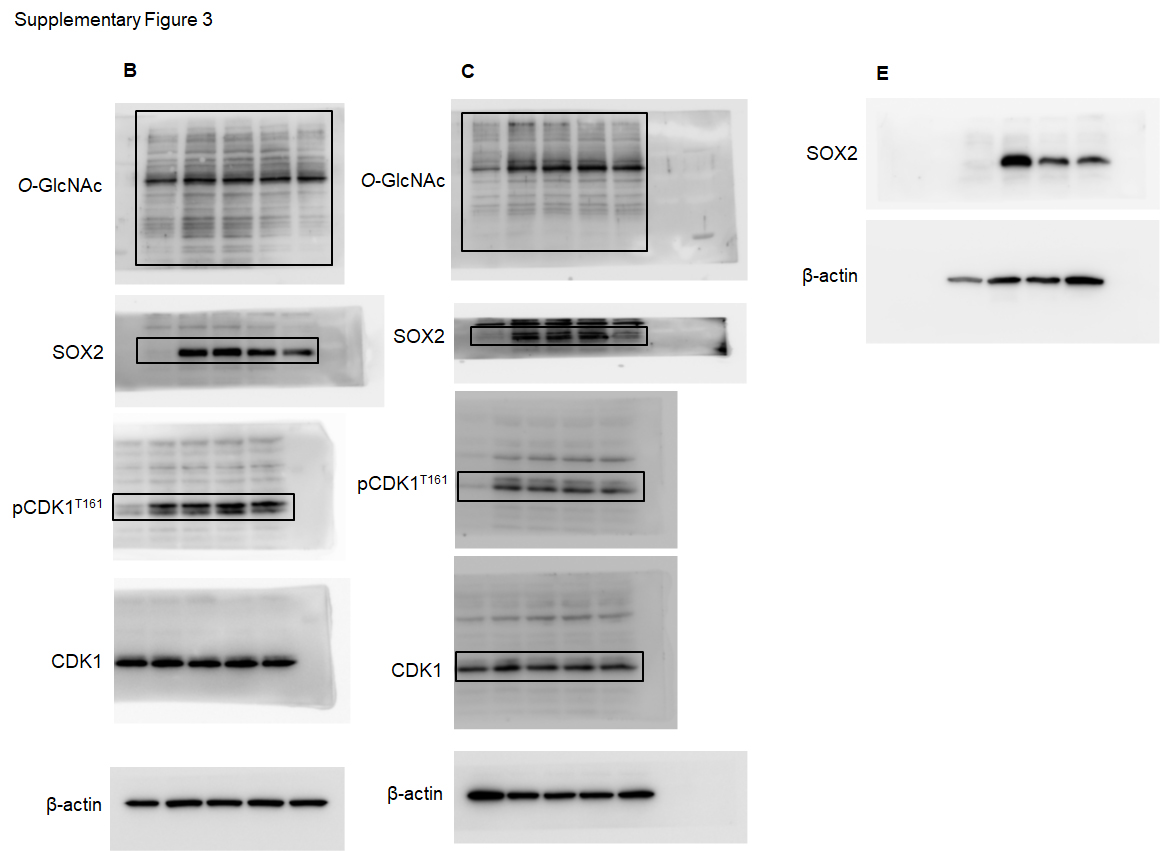


**Supplementary Figure 5 continued**


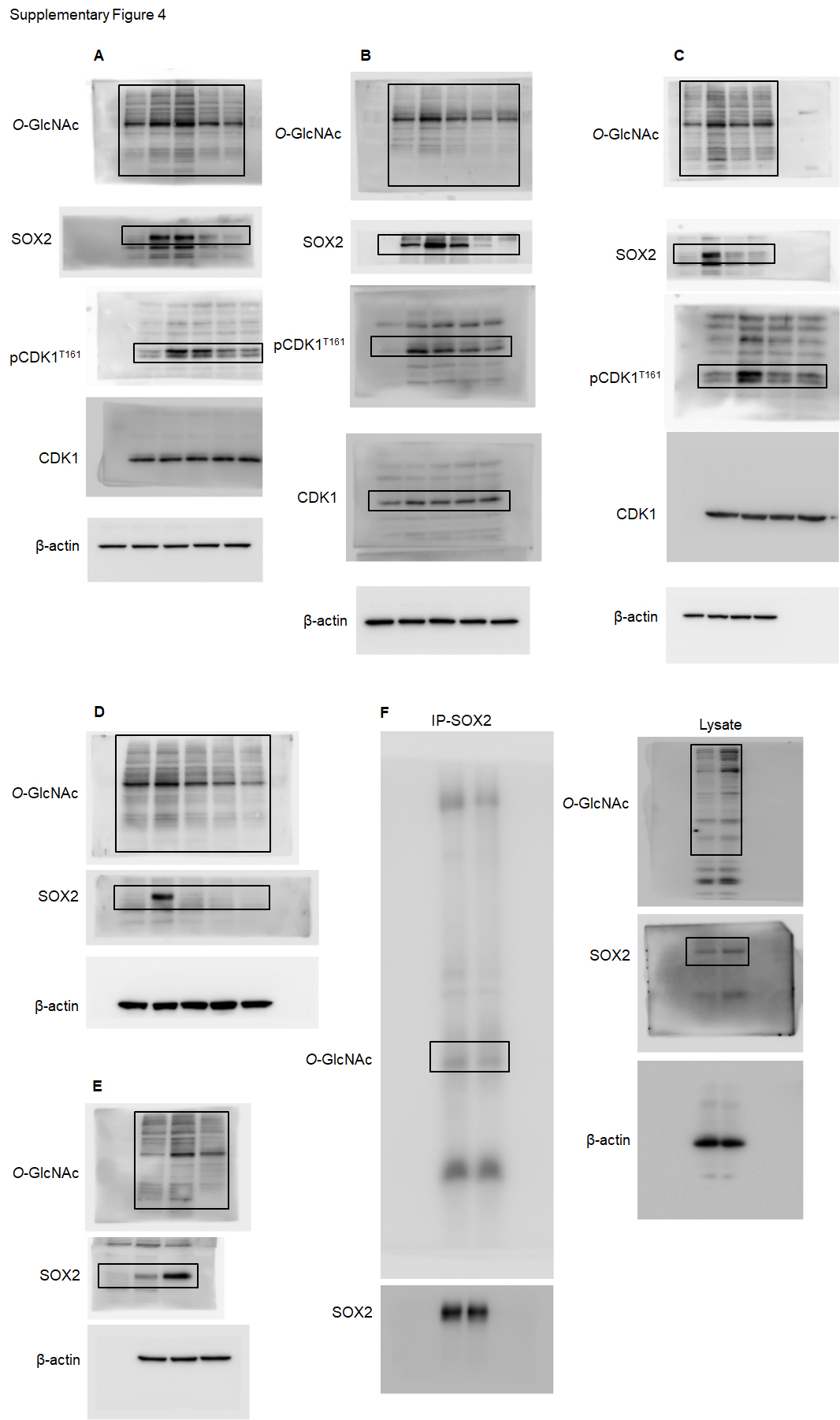

Supplement: Supplementary file 1 — Supplementary Information. [file 41598_2022_6916_MOESM1_ESM.docx]
